# Efficient signed-rank based EWMA and HWMA repetitive control charts for monitoring process mean with and without auxiliary information

**DOI:** 10.1038/s41598-023-42632-x

**Published:** 2023-09-30

**Authors:** Ambreen Shafqat, Huang Zhensheng, Muhammad Aslam

**Affiliations:** 1 Department of Financial Mathematics and Statistics, Nanjing University of Science and Technology, Nanjing, 210094 China; 2 Department of Statistics, Faculty of Science, King Abdulaziz University, 21551 Jeddah, Saudi Arabia; 3 Department of Urology, Roswell Park Cancer Research Institute, Buffalo, NY 14263 USA

**Keywords:** Engineering, Mathematics and computing

## Abstract

Control charts are powerful tools to observe the presentation of the manufacturing process. Mostly, when the data in industries come from the process may follow non-normal or unknown distributions. So, the distribution-free control charts are useful in practice when the possibility model of the process productivity is unknown. In such situations, the correct selection of the sampling mechanism is beneficial for process examination. This paper proposes a nonparametric exponentially weighted moving average signed-rank (EWMA-SR) and also proposed a homogeneously weighted moving average Signed-Rank (HWMA-SR) control charts for examining the small shift in process with the help of an auxiliary variable (in the form of a regression estimator) by using an efficient plan, namely, a repetitive sampling plan. The proposal’s presentation is evaluated and matched with its complements for different symmetric distributions by using some famous run length properties including average run length, median run length, and standard deviation of run length.

## Introduction

The quality of the product is an essential factor for keeping the attractiveness of industries and is also significantly affected by the stability of the product manufacturing process. A quality manager uses different management tricks and engineering methods to make a valuable quality product. In some situations, the product is physically good whereas in others it maybe not. Industries need to assemble always good quality products that fulfill the client’s requirements. The clients’ satisfaction should be the primary concern because if the products’ quality is not according to the clients’ need the company can’t easily sell the kinds of stuff no matter what its price is. In the worst conditions, the clients may take their business anywhere else. This is when statistical process control (SPC) comes into action and handles the company business with the presentation of control charts. He and Wang^1^ first time proposed a roadmap of statistical process monitoring (SPM) which divided the development of SPM into three generations: The first generation of the SPM is SPC: 2nd generation is multivariate statistical process monitoring (MSPM): and 3rd generation: yet to be appropriately defined and named. So the SPC control charts are extensively expended tools in SPM for examining and purifying the procedure quality. The companies can easily monitor their business with the control charts application and manufacture quality products. Basically, the control chart helps to ascertain the natural and unnatural variations, as a result, makes less production of defective items. There are two types of control charts first one is memoryless control charts (Phase I) and the second type of control chart is memory type control charts (Phase II), which utilized the current information plus previous information. A lot of memory-type and memoryless control charts are available in the literature that is best to use for getting quality products in manufacturing industries such as Abbas et al.^2^ introduced a progressive mean control chart to monitor the fraction nonconforming, Nazir et al.^3^ proposed the mixed memory-type control chart, Aslam et al.^4^ developed a control chart for Birnbaum-Saunders distribution by using the multiple dependent state repetitive sampling and Shafqat et al.^5,6^ designed a parametric memoryless control chart by using the repetitive sampling scheme.

Generally, the data used in SPM methods are strained since the normal distribution, but when important features break the assumption of normality, then SPM methods may flop or produce defective items. In many industrial processes, the characteristics of service quality have no idea about distribution. So, using a parametric control chart to display the process dissimilarities is not a good decision. In this case, the nonparametric methods have been used to acquire the control charts for procedure checking for non-normal data observations. Anad et al.^7^ introduced a new nonparametric control chart that is the combination of the Shewhart and the classical EWMA chart in a smooth way and shows this chart performance is best. Other than that, a lot of research is also conducted for the nonparametric or distribution-free process such as Graham et al.^8^ introduced distribution-free EWMA charts by using the two nonparametric test statistics, namely sign and signed-rank. Chakraborti and Van de Wiel^9^, Zhou et al.^10^, and Hawkins and Deng^11^ used the well-known nonparametric Mann-Whitney test statistic to develop control charts to detect location shifts. Shih-Chung^12^ designed a new framework for nonparametric profile monitoring. Wang et al.^13^ designed a distribution-free control chart by using the influential nonparametric likelihood ratio goodness of fit test with an efficient change-point model to notice any variation in the distribution. Zou et al.^14^ developed a distribution-free EWMA control chart based on the likelihood ratio. In Ref.^15^ designed significant research on nonparametric regression with the generalized likelihood ratio chart, $T^2$ chart, and EWMA chart for the profile monitoring with attribute data. Li et al.^16^ proposed a signed-rank constructed nonparametric control chart for examining the scale and location parameters of the univariate continuous development distribution. Abid et al.^17^ also used the ranked set sampling plan to design distribution-free EWMA charts using the sign test statistics for observing the process location. Moreover, Graham et al.^18^ introduced a nonparametric Phase-II EWMA control chart based on an exceedance test statistic for observing procedures with an unknown location parameter. The HWMA control chart introduced by^19^ is a memory-type chart proposed for well-organized monitoring of small to moderate shifts ($\delta \le 0.5$ to $0.5< \delta < 2$) in the process mean. Other memory-type charts include the EWMA, the CUSUM chart, and the mixed EWMA-CUSUM charts proposed by^20^. Moreover, new distribution-free HWMA control charts have been introduced by^21^ using the two nonparametric tests, namely, Sign and Signed-rank test statistics for the process location of the target value. Recently, Abbas et al.^22^ introduced a nonparametric progressive sign-based control chart for individual data. A control chart based on artificial neural networks (ANN) is developed to track the linear profile in phase II of the process quality using machine learning techniques to monitor the relationship between the response variable and one or more independent variables^23^. A wavelet-based nonparametric CUSUM chart based on adaptive thresholding designed for high dimensional profile components for randomly sampled phase I profile to monitor Hoteling’s type statistics^24^. The run rule in statistical process monitoring based control chart designed for the detection of different shift sizes, especially small and medium shifts^25^.

In recent years, the researchers have started studying the existing control charts, like the Shewhart, EWMA, CUSUM, and mixed control charts, etc., with the help of new well-organized mean or variance estimators that involve information not only on the study variable but also on the linked auxiliary variables. Using data on different quality-related characteristics is a standard preparation in the multivariate SPC. The use of appropriate supplementary information helps in amassing the exactness of an estimator. Hence, the efficient estimators-based control charts turn out to be more sensitive or efficient. Following these ideas, Nurudeen et al.^26^ developed an HWMA chart that customs the auxiliary and process variables in the procedure of a regression estimator to require an unbiased and efficient assessment of the process’s mean variables, namely, AHWMA. In the case of normality, the AHWMA chart has efficient performance in shift revealing power as compared to classical charts such as EWMA, CUSUM, HWMA^27^, auxiliary-based EWMA chart^28^ and the auxiliary-based CUSUM chart^29^. Other researchers have reviewed and suggested auxiliary-based control charts in the process variables monitoring for normal distribution and designed different control chart methods. As Mandel^30^ proposed a regression control chart with an auxiliary variable. At the same time, Zhang^31^ introduced a cause-selecting control chart. Raiz et al.^32^ designed a Shewhart-type chart with an auxiliary variable, namely, Vr chart, for monitoring process variability. Furthermore, Raiz et al. worked and proposed different EWMA and CUSUM-type control charts based on the auxiliary information for monitoring location and the process mean (see^33^).

After exploring the literature related to control charts, it is noticed that most control charts are designed for a single sampling scheme. Some researchers examined more well-organized sampling schemes for different types of control charts such as simple random sampling (SRS), double sampling (DS), multiple dependent states (MDS), repetitive sampling (RS), ranked set sampling (RSS), and sequential sampling (SS) scheme. Abbas et al.^34^ decorated a more efficient, easy, and distribution-free control chart under a simple and ranked set sampling scheme. Aslam et al.^35^ used the MDS scheme and developed the ${\overline{X}}$ control chart. Raiz et al.^36^ introduced a nonparametric sign test-based control chart under a sequential sampling scheme. Some nonparametric control charts are introduced based on location, shifts, and dispersion. Recently, some researchers such as Ali et al.^37^, Abid et al.^38^, and Celano et al.^39^ used different nonparametric tests and various sampling schemes for designing the control chart (Table 1).

Some dissimilar methods to change the sampling rate, such as the variable sampling interval and variable sample size and sampling interval. Reynolds and Arnold^40^ designed the EWMA control chart with the variable sample size and variable sampling intervals features. Li and Qiu^41^ also utilized the sampling interval task for the p-value of the CUSUM charting statistic, called a dynamic sampling scheme. Furthermore, the repetitive sampling (RS) plan is another scheme that is very important nowadays in the SPC literature. The RS scheme chart for method examining is created on compelling samples of a specific size from the process through a specified sampling interval. The first time, Sherman^42^ introduced the RS plan and primary single sampling and reviewed its pros: for example, it is more efficient in detection as compared to single and double sampling schemes and more comfortable to apply than sequential sampling (SS). Meanwhile, the RS scheme’s usage converts more admired in the control chart area due to its efficiency. The RS scheme method is similar to the SS scheme, but this scheme provides the necessary protection to the consumers and the producer apart from the smallest sample size. In RS, a sample is selected from the industrial process to determine the control chart’s condition. In the case of non-decision, the second sample is selected, and the process of selecting the sample is continued until the decision is reached. Some of the latest works in nonparametric RS direction are those of Aslam et al.^43^, Azam et al.^44^, Shafqat et al.^45^, Chen et al.^46^, and Jean-Claude et al.^47^. They show that RS scheme performance is better in detecting the out-of-control values comparatively small process mean shifts as compared to single and double sampling.

This manuscript proposes an auxiliary-based HWMA control chart for monitoring changes in the process mean. This work is the first step in the nonparametric control chart construction of auxiliary-based information. With the help of the Monte Carlo simulation method, the run-length characteristics, which include the average run-length and standard deviation of run-length are calculated. Conversely, in the non-normal distributions, the IC ARL of the homogeneously weighted moving average control chart using the auxiliary information (regression-based estimator) deflates under the heavily tailed and skewed distributions (like student’s t, logistic, and Laplace distribution). Besides that, determined by the rewards of the RS scheme and properties of EWMA, HWMA, and auxiliary HWMA signed-rank charts, this paper also attempts to extend the nonparametric EWMA signed-rank chart of Graham et al.^19^ and HWMA control chart of Raza et al.^26^ to the nonparametric signed-rank control charts using repetitive sampling, namely, EWMA-SR repetitive and HWMA-SR repetitive. Moreover, a new HWMA-SR repetitive chart based on an Auxiliary variable for monitoring process location under different distributions is also proposed in this paper. The proposed charts are performed best for shift detection as compared to the existing control charts. Chen JH et al.^48–51^ worked on EWMA-t, HWMA, and AHWMA charts with various techniques and proved the efficiency of the proposed charts. Noor ul Amin et al.^52^ developed the HWMA chart with Auxiliary information for the average-variance chart. The coefficients for control charts in repetitive sampling for in-control processes are determined when the average sample size over time ($ASS_0$) is equal to $'n'$. In this scenario, the average run lengths ($ARL_1$) are smaller compared to the Shewhart control chart. It’s worth noting that in cases where repetitive sampling is employed with a sample size equal to $ASS_0$, it may not necessarily be the same as $ASN_1$, especially when dealing with various shifts. Furthermore, the authors suggest that when ASS1 is in proximity to $'n'$, increasing the sample size in the Shewhart control chart can lead to improved efficiency. However, the authors did not provide a direct comparison at identical sample sizes. The approach of gauging efficiency solely by increasing the sample size is deemed flawed. In essence, the work by Saleh et al.^53^ presents a comparison that appears illogical, as it overlooks the assumptions inherent to repetitive sampling and seems to disregard the optimization methods outlined in existing literature. Upon reviewing the study by Saleh et al.^53^, it becomes evident that their primary intention was to criticize the applicability of repetitive sampling in future scenarios, potentially undermining its practical use.

**Table 1:** Summary of the literature on HWMA-related monitoring schemes.

*Paper*	Scheme	Assumption	Characteristic	Parameter(s)	Data	Process	Chart’s scheme
Abbas (2018)	Variable	Parametric	Location	Known	Univariate	I.I.D	HWMA${\overline{X}}$
Adegoke et al. (2019)	Variable	Parameter	Location	Known	Univariate	Serial D.	HWMA ${\overline{X}}$ with AIB
Riaz et al. (2020)	Variable	Parametric	Variability	Known	Univariate	I.I.D	HWMA *S*
Dawod et al. (2020)	Variable	Nonparametric	Profile	Unknown	Multivariate	Serial D.	HWMA Profile
Noor-ul et al. (2021)	Variable	Parametric	Variability	Known	Univariate	I.I.D	AHWMA*S*
Lu SL. et al. (2021)	Variable	Parametric	Location	Known	Univariate	I.I.D	AGWMA${\overline{X}}$
Alevizakos et al. (2021)	Variable	Nonparametric	Location	Known	Univariate	I.I.D	DHWMA*SN*
Raiz et al. (2021)	Variable	Parametric	Location	Known	Univariate	I.I.D	THWMA
Letshedi et al. (2022)	Variable	Nonparametric	Location	Known	Univariate	I.I.D	HWMA
Naveed et al. (2023)	Variable	Parametric	Location	Known	Univariate	I.I.D	Ex.EWMA with AIB

Know et al.^54^ “HWMA chart loses its performance as compared to EWMA chart in steady-state”. The re-investigated the performance of the HWMA chart under zero and steady states at various shifts. According to Riaz et al.,^55,56^ “A comprehensive comparative analysis of the run-length profiles is carried out among the two charts for several values of the design parameters. The results revealed that the HWMA chart is superior to the EWMA chart under a zero state for several regions of shifts, and is capable of retaining its superiority over EWMA under various delays in process shifts. More specifically, the steady-state performance of every moving average control chart depends on the choice of the design parameter. This study has identified the dominance cut-offs for HWMA and EWMA. We noticed that both EWMA and HWMA have their respective superiority regions depending on the choices of parameters”.

The rest of the paper is divided as follows. The existing nonparametric control chart structures are reviewed in Section "Introduction". The proposed chart design structures are reviewed in Section "Some existing nonparametric signed-sank (SR) control charts structures". The performance evaluation of the proposed charts with results discussion and comparison of proposed charts with their counterparts are mentioned in Section "The design of all proposed nonparametric signed-rank repetitive control charts". The Illustrative Example detail of the proposed vis existing charts is described in Section  "Performance evaluation of proposed charts" and the Decision plus future recommendations of the plotting charts are in Section  "Performance evaluation of proposed charts".

## Some existing nonparametric signed-sank (SR) control charts structures

In this section, the designs of memory-type control charts (EWMA-SR, HWMA-SR, and Auxiliary HWMA-SR) are presented, which are used for the detection of small to moderate and moderate to large process shifts.

### The structure of nonparametric EWMA-SR chart

Let suppose that $X_{ij}$, $i=1,2,3,...n$ and $j=1,2,....$ denote the $i{th}$ observation in the $j{th}$ subgroup of size $n>1$. $R_{ij}^{+}$ is the rank of the absolute values of the differences $|X_{ij}-\theta _0 |, i=1,2,....n$ within the $j{th}$ subgroup. Define as:

1 $$\begin{aligned} SR_{Xj}=\Sigma _{i=1}^n A_{ij}R_{ij}^+, j=1,2,3,...,... \end{aligned}$$

where

$$\begin{aligned} A_{ij} = {\left\{ \begin{array}{ll} 1, \quad \text{ if } (X_{ij}-\theta _0) >0\\ 0, \quad \text{ if } (X_{ij}-\theta _0)=0\\ -1, \quad \text{ if } (X_{ij}-\theta _0) <0\\ \end{array}\right. } \end{aligned}$$

and $\theta _0$ is the well-known target value of the process. So, $SR_{Xj}$ is the difference between the sum of the rank of the absolute differences corresponding to the positive and the negative differences. The $SR_{Xj}$ is linearly related to the better-known Mann-Whitney test statistic $T_{n}^{+}$ through the relationship $SR=2T_n^+ - n(n+1)/2$ (See^38^ for more detail about the Mann-Whitney test statistic). The mean and variance of the *SR* is zero and $n(n+1)(2n+1)/6$, respectively. The nonparametric EWMA-SR^39^ control chart statistic is stated as:

2 $$\begin{aligned} Z_j=\omega SR_{Xj} + (1-\omega )Z_{j-1} \end{aligned}$$

Here smoothing constant $\omega$ lies between 0 and 1 i.e., ($0<\omega \le 1$) and $Z_j$ is identified as smoothed values and $Z_0=\theta _0$. The mean and variance of the EWMA-SR chart are:

3 $$\begin{aligned} E(Z_j)= & {} \theta _0=0\nonumber \\ Var(Z_j)= & {} \frac{\omega ((1-(1-\omega )^{2i})n(n+1)(2n+1)}{(2-\omega )6} \end{aligned}$$

The asymptotic control limits of nonparametric EWMA-SR chart for single sampling method when *i* has largest values and approaches to 1. It can be written as:

$$\begin{aligned} UCL/LCL=\theta _0 \pm L \sqrt{\frac{\omega n(n+1)(2n+1)}{(2-\omega )6}} \end{aligned}$$

### The structure of nonparametric HWMA-SR statistic

The HWMA-SR chart is constructed by calculating the statistics $SR_1, SR_2, SR_3,....$ sequentially from each subgroup. The plotting statistic is defined as:

4 $$\begin{aligned} H_j=\omega {SR}_{Xj}+(1-\omega ) {\overline{SR}}_{X(j-1)} \end{aligned}$$

Where ${SR}_{X(j-1)}$ is the sample mean for $(j-1)$ sample and $\omega$ is the smoothing constant which is called the sensitivity parameter of the HWMA statistic i.e. $0< \omega \le 1$ is given as.

$$\begin{aligned} {\overline{SR}}_{X(j-1)}=\frac{\Sigma _{k=1}^{j-1}{SR}_{Xk}}{j-1} \end{aligned}$$

The HWMA-SR statistic (Eq. 4) mean is $\mu _{SR}=\mu _H=\theta _0=0$, and variance can be derived as detailed can be seen in online Appendix A.1.

5 $$\begin{aligned} \sigma _{H_j}^2= {\left\{ \begin{array}{ll} \frac{\omega ^2 n(n+1)(2n+1)}{6}, \quad \text{ if } j=1\\ \frac{n(n+1)(2n+1)}{6} (\omega ^2+\frac{(1-\omega )^2}{j-1}), \quad \text{ if } j>1\\ \end{array}\right. } \end{aligned}$$

So the control limits of the HWMA-SR chart for single sampling method are:

6 $$\begin{aligned} LCL_j= & {} {\left\{ \begin{array}{ll} \theta _0 - k \sqrt{{\frac{\omega ^2 n(n+1)(2n+1)}{6}}} \quad \text{ if } j=1\\ \theta _0 - k \sqrt{{\frac{ n(n+1)(2n+1)}{6} (\omega ^2+\frac{(1-\omega )^2}{j-1})}} \quad \text{ if } j>1 \end{array}\right. } \end{aligned}$$

7 $$\begin{aligned} UCL_j= & {} {\left\{ \begin{array}{ll} \theta _0 +k \sqrt{ \frac{\omega ^2 n(n+1)(2n+1)}{6}} \quad \text{ if } j=1\\ \theta _0 +k \sqrt{ \frac{ n(n+1)(2n+1)}{6} (\omega ^2+\frac{(1-\omega )^2}{j-1})} \quad \text{ if } j > 1 \end{array}\right. } \end{aligned}$$

Where *k* is the coefficient of the control limit and is used for calculating the width of the control limits based on the desired $ARL_0$.

### The structure of nonparametric auxiliary HWMA-SR statistic

In the SPC literature, the sensitivity of a control chart increases by selecting a more precise estimator of the population parameter, i.e., the better the estimator, the better will be the compassion of a control chart. In what follows, we propose an auxiliary HWMA-RS control chart for monitoring the process mean^48^. Suppose that there is another auxiliary variable, $Y_{ij}$ that is linked with the main variable of relevance, $X_{ij}$ with association $\rho$. We undertake the observations of (*X*, *Y*) are perceived in pairs from a bivariate normal distribution, given by $(X,Y)\sim N_2 (\mu _X,\mu _Y, \sigma _X, \sigma _Y, \rho )$ and can be modeled the linear relationship between variables using least squares getting by modifying the mean of the process at time $\mu _{X}$ shows the known relationship with new variable. For this purpose, an unbiased estimator of the process $\mu _X$, say $Y^*$, is given by.

8 $$\begin{aligned} Y^*_j=X+b (\mu _{Y} -Y) \end{aligned}$$

with its mean and variance given by

9 $$\begin{aligned} E(Y^*)=\mu _{Y*}, V(Y^*)=\sigma ^2_{X}(1-\rho ^2) \end{aligned}$$

Here *b* is a regression line slop which is actually a variation in the procedure variable X due to the unit change in the auxiliary variable $Y, (b=\frac{\rho \sigma _X }{\sigma _Y})$, detail can be seen in^27^. Respectively, the mathematical expectation and variance with respect to the random variable *Y* and $Y^*$ is a more precise variable than *Y* is also unbiased. Due to this efficiency of a new variable, we propose an auxiliary HWMA signed-ranked chart for monitoring the process mean. For monitoring the process mean $\mu _X$, assume that a bivariate simple random sample of size *n*, denoted by $(X_{ij}, Y_{ij})$. So, the $R_{ij}^{+}$ is the rank of the absolute values of the differences $|Y^*_{ij} - \theta _0|$, $i=1,2,....,n$ within the $j{th}$ subgroup. Define as:

10 $$\begin{aligned} SR_{Y^*_j}=\Sigma _{i=1}^n A^*_{ij} R_{ij}^+ \end{aligned}$$

where

$$\begin{aligned} A^*_{ij} = {\left\{ \begin{array}{ll} 1, \quad \text{ if } (Y^*_{ij}-\theta _0) >0\\ 0, \quad \text{ if } (Y^*_{ij}-\theta _0)=0\\ -1, \quad \text{ if } (Y^*_{ij}-\theta _0) <0\\ \end{array}\right. } \end{aligned}$$

The mean and variance of $SR_{Y^*_j}$ given by:

$$\begin{aligned} \mu _{Y}=\mu _{SR_{X_j}}=\mu _{SR_{Y^*_j}}=\mu _{SR}=\mu _H \\ \sigma _{SR_{Y^*}}=\sigma _{SR}^2 =\frac{n(n+1)(2n+1)}{6} \end{aligned}$$

The plotting statistic of the Auxiliary HWMA control chart can be written as below by using the Eq. (10):

11 $$\begin{aligned} M_j=\omega SR_{Y^*_j} +(1-\omega ) {\overline{SR}}_{Y^*( j-1)} \end{aligned}$$

where $\omega$ $(0\le \omega \le 1)$ is the smoothing parameter, $SR_{Y^*_j}$ can be regarded as the difference between the sum of the ranks of absolute difference corresponding to the $+ve$ and $-ve$ differences based on a regression-informed estimate which can be calculated by using the Eq. (10), and ${\overline{SR}}_{Y^*(j-1)}$ is the average of all the previous sample mean of the scheming statistic. The ${\overline{SR}}_{Y^*(j-1)}$ is determined as ${\overline{SR}}_{Y^*(j-1)}=\frac{1}{j-1} \Sigma _{i=1}^{j-1}SR_{Y^*_i}$ and the mean of the plotting statistic is $\mu _M=\mu _{SR}=\mu _H$, and variance is:

12 $$\begin{aligned} \sigma _{M_j}^2= {\left\{ \begin{array}{ll} \omega ^2 \sigma _{SR}^2, \quad \text{ if } j=1\\ (\omega ^2 \sigma _{SR}^2+\frac{(1-\omega ^2)\sigma _{SR}^2}{j-1}) \quad \text{ if } j>1\\ \end{array}\right. } \end{aligned}$$

The control limits for the plotting statistic under single sampling are given as:

13 $$\begin{aligned} LCL_j= & {} {\left\{ \begin{array}{ll} \theta _0 - k \sqrt{ \omega ^2 \sigma _{SR}^2} \quad \text{ if } j=1\\ \theta _0 - k \sqrt{(\omega ^2 \sigma _{SR}^2+\frac{(1-\omega ^2)\sigma _{SR}^2}{j-1})} \quad \text{ if } j>1 \end{array}\right. } \end{aligned}$$

14 $$\begin{aligned} UCL_j= & {} {\left\{ \begin{array}{ll} \theta _0 +k \sqrt{ \omega ^2 \sigma _{SR}^2} \quad \text{ if } j=1\\ \theta _0 +k \sqrt{ (\omega ^2 \sigma _{SR}^2+\frac{(1-\omega ^2)\sigma _{SR}^2}{j-1})} \quad \text{ if } j>1 \end{array}\right. } \end{aligned}$$

where *k* is the control limits constant which is used to determine the width of control limits from the central line to lower and upper limits and decided according to the fixed in-control ARL values.

## The design of all proposed nonparametric signed-rank repetitive control charts

**Figure 1:**
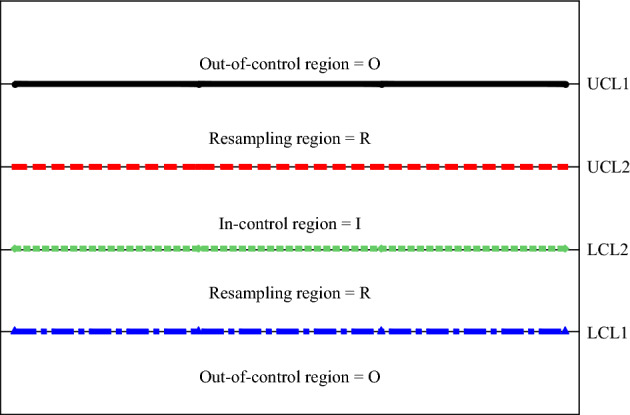
Control chart with repetitive sampling structure.

Here, provides the design of EWMA-SR, HWMA-SR, and Auxiliary HWMA-SR control charts by using a repetitive sampling (RS) scheme. The RS plan is created on two outer and two inner control limits. Between the outer control limits, observations resolve the process is out of control while the inner control limits decide the process is in control. But if the observations are inside between the two inner and two outer control limits, it determines the repetitive part (see Fig. 1).

### The proposed nonparametric EWMA-SR repetitive control chart

The proposed nonparametric EWMA-SR repetitive control chart follows these steps:

Step 1. Select a simple random sample from the production process and smoothing parameters ($\omega$) for the EWMA-SR control chart.

Step 2. Calculate the test Statistic $SR_{Xj}$ from the fixed distribution studied in this study and subsequently compute the charting statistic $Z_j$ using Eq. (2).

Step 3. Find upper and lower control limits as follow:

$$\begin{aligned} UCL_1/LCL_1&= \pm k_1 \sqrt{\frac{\omega n(n+1)(2n+1)}{(2-\omega )6}}\\ UCL_2/LCL_2&= \pm k_2 \sqrt{\frac{\omega n(n+1)(2n+1)}{(2-\omega )6}} \end{aligned}$$

where $k_1$ and $k_2$ are control limit coefficients ($k_1>k_2$)^57^ and select the values of coefficients for a specified $ARL_0$.

Step 4. Compute the control limits and compare the charting statistics with control limits as if $LCL_2\le Z_j \le UCL_2$, the process declared in-control and if $Z_j < LCL_1 or Z_j > UCL_1$, then the process is declared out of control. But if $LCL_1 \le Z_j <LCL_2$ or $UCL_2<Z_j \le UCL_1$, then go to step 1. and repeat the process until a decision is out of control with their individual control limits.

Step 5. Trace the subgroup number until the plotting statistic declared OOC observation the first time.

### The proposed nonparametric HWMA-SR repetitive control chart

The proposed nonparametric HWMA-SR repetitive control chart follows these steps:

Step 1. Select a simple random sample from the production process and smoothing parameters ($\omega$) for the HWMA-SR control chart

Step 2. Calculate the test Statistic $SR_{X_j}$ and ${\overline{SR}}_{X_(j-1)}$ from the exact distribution studied in this study and subsequently compute the plotting statistic $H_j$ using Eq. (4).

Step 3. Find upper and lower control limits as follow:

$$\begin{aligned} UCL_1/LCL_1&=\pm k_1 \sqrt{\sigma _{H_j}^2}\\ UCL_2/LCL_2&=\pm k_2 \sqrt{\sigma _{H_j}^2} \end{aligned}$$

where $k_1$ and $k_2$ are control limit coefficients ($k_1>k_2$)^5^. In Eq. (7) if $j \rightarrow \infty$ the variance of the charting statistic ($H_j$) approaches to $\frac{(\omega ^2 (n(n+1)(2n+1)))}{6}$. So, the proposed HWMA-SR repetitive chart control limits are given as the following:

$$\begin{aligned} UCL_1/LCL_1&=\pm k_1 \sqrt{\frac{\omega ^2n(n+1)(2n+1)}{6}}\\ UCL_2/LCL_2&=\pm k_2 \sqrt{\frac{\omega ^2n(n+1)(2n+1)}{6}} \end{aligned}$$

Step 4. if $LCL_2\le H_j\le UCL_2$, the process is declared in control, and if $H_j < LCL_1$ or $H_j > UCL_1$, then the process is declared out of control. But if $LCL_1\le H_j < LCL_2$ or $UCL_2 < H_j\le UCL_1$, then go to step 1. and repeat the process until a decision is out of control. The charting parameters, $k_1, k_2$ and $\omega$ for EWMA-SR repetitive chart and $k_1,k_2$ and $\omega$ for HWMA-SR repetitive chart are selected in such a way that an assured $ARL_0$ got for a specified sample size (*n*). So the fixed value of IC ARL for EWMA-SR repetitive chart and HWMA-SR repetitive chart depends on the values of designed parameters, i.e., $ARL_0=f(n, k_1, k_2,\omega )$ and $ARL_0=f(n, k_1, k_2,\omega )$. Some selections of the designed parameters for both the plotted charts for various values of n under nominal $ARL_0$ values of 370 and 500 are offered in Table 2.

**Table 2:** Combination of design parameters of EWMA-SR repetitive and HWMA-SR repetitive (first row contain EWMA-SR and 2nd row contain HWMA-SR) control charts to attain nominal $ARL_0=370,500$.

$Nominal ARL_0=370$		$Nominal ARL_0=500$	
(coefficients)	Attained	(coefficients)	Attained
($k_1, k_2$)	$ARL_0$	$(k_1, k_2)$	$ARL_0$
n=5,$\omega =0.03$			
(2.479,0.893)	370.2	(2.478,1.527)	500.3
(2.085,0.414)	370.4	(2.289,0.451)	500.5
n=5,$\omega =0.05$			
(2.645,0.887)	370.7	(2.641,1.487)	501.2
(2.155,0.975)	370.8	(2.279,0.533)	500.2
n=10,$\omega =0.03$			
(2.528,0.782)	370.2	(2.576,0.989)	501.8
(2.334,0.470)	370.7	(2.389,0.554)	500.7
n=10,$\omega =0.05$			
(2.596,1.109)	370.1	(2.625,1.712)	500.5
(2.544,0.685)	370.4	(2.677,0.525)	501.5
n=10,$\omega =0.10$			
(2.739,1.399)	370.4	(2.809,1.829)	500.1
(2.650,1.190)	370.4	(2.797,0.619)	500.3
n=10,$\omega =0.20$			
(2.827,1.729)	370.1	(2.927,1.603)	501.5
(2.799,1.279)	371.7	(2.816,0.557)	501.2
n=15,$\omega =0.03$			
(2.526,0.769)	371.2	(2.537,1.138)	500.4
(2.497,0.409)	370.6	(2.630,0.491)	500.8
n=15,$\omega =0.05$			
(2.607,1.079)	371.3	(2.697,1.199)	501.1
(2.537,0.791)	370.4	(2.755,0.638)	501.5
n=15,$\omega =0.10$			
(2.794,1.104)	370.0	(2.867,1.289)	500.9
(2.837,0.866)	370.1	(2.955,1.396	501.8
n=15,$\omega =0.20$			
(2.889,1.204)	370.1	(2.968,1.332)	500.5
(2.939,1.496)	370.1	(3.055,0.846)	500.9
n=20,$\omega =0.03$			
(2.553,0.727)	370.1	(2.469,1.497)	501.9
(2.443,0.398)	370.5	(2.455,0.494)	500.8
n=20,$\omega =0.05$			
(2.519,1.485)	370.6	(2.669,1.397)	500.5
(2.535,0.746)	370.4	(2.698,0.549)	501.6
n=20,$\omega =0.10$			
(2.729,1.574)	370.2	(2.829,1.745)	501.6
(2.685,0.973	370.4	(2.798,0.895)	501.8
n=20,$\omega =0.20$			
(2.869,1.434)	370.3	(2.965,1.634)	500.3
(2.885,0.995)	370.6	(2.998,0.916)	500.6

### The proposed nonparametric auxiliary HWMA-SR repetitive control chart

The proposed nonparametric Auxiliary HWMA-SR repetitive control chart follows these steps:

Step 1. Select a simple random sample from the production process and smoothing parameters ($\omega$) for the Auxiliary HWMA-SR control chart.

Step 2. Calculate the plotting Statistic $M_j, SR_{Y^*_j}$ and ${\overline{SR}}_{Y^*(j-1)}$ from the specific distribution considered in this study and subsequently compute the charting statistic $M_j$ using Eqs. 10 and 11.

Step 3. Find upper and lower control limits as follow:

$$\begin{aligned} UCL_1/LCL_1&=\pm k_1 \sqrt{\sigma _{M_j}^2}\\ UCL_2/LCL_2&=\pm k_2 \sqrt{\sigma _{M_j}^2} \end{aligned}$$

where $k_1$ and $k_2$ are control limit coefficients ($k_1 > k_2$). In Eq. (11) if $j\rightarrow \infty$ the variance of the charting statistic ($M_j$) approaches to $\frac{\omega ^2 (n(n+1)(2n+1))}{6}$. So, the proposed chart HWMA-SR repetitive control limits are given as the following:

$$\begin{aligned} UCL_1/LCL_1&=\pm k_1 \sqrt{\frac{\omega ^2 (n(n+1)(2n+1))}{6}}\\ UCL_2/LCL_2&=\pm k_2 \sqrt{\frac{\omega ^2 (n(n+1)(2n+1))}{6}} \end{aligned}$$

Step 4. If $LCL_2\le M_j\le UCL_2$, the process is declared in control, and if $M_j<LCL_1$ or $M_j>UCL_1$, then the process is declared out of control. But if $LCL_1\le M_j<LCL_2$ or $UCL_2<M_j\le UCL_1$, then go to step 1. and repeat the process until a decision is out of control. The designed parameters $(k_1,k_2, \omega , \rho )$ of the Auxiliary HWMA-SR repetitive chart are selected in such a way that assured the value of $ARL_0$ is got for a fixed value of sample size (*n*). So, the $ARL_0$ of the planned chart is contingent on the values of designed parameters, i.e., $ARL_0=f(n,\omega ,\rho , k_1, k_2)$. Some possible values of the designed parameters of the proposed chart for the different subgroup sizes under nominal $ARL_0=370, 500$ are listed in Table 3. During the calculation, it has been seen that when $n\le 5$ and $\omega \ge 0.10$ the nominal $ARL_0=370, 500$ are not attainable.

**Table 3:** The $k_1, k_2$ values of the proposed Auxiliary HWMA-SR repetitive control chart to attain nominal $ARL_0 = 370, 500$.

$\omega$	$\rho$			
	0.05	0.25	0.5	0.75
	$$ARL_0=370$$			
$$n=5$$				
0.03	(2.25, 0.43)	(2.45, 0.47)	(2.67, 0.49)	(3.92, 1.27)
0.05	(2.28, 0.35)	(2.53, 0.41)	(2.89, 0.81)	(4.29, 2.74)
$$n=10$$				
0.03	(2.51,0.38)	(2.68,0.48)	(2.59,1.29)	(4.39,1.95)
0.05	(2.74, 0.39)	(2.67,0.76)	(3.09, 1.50)	(4.76, 2.60)
0.10	(2.82, 0.47)	(3.202, 1.152)	(3.50, 2.14)	(4.98, 3.94)
0.20	(2.85, 0.59)	(3.09, 1.89)	(3.78, 2.28)	(5.38, 2.17)
$$n=15$$				
0.03	(2.36, 0.37)	(2.50, 0.62)	(2.86, 1.58)	(4.07, 2.23)
0.05	(2.68, 0.41)	(2.66, 0.91)	(3.33, 1.58)	(4.57, 2.41)
0.10	(2.93, 0.67)	(3.09, 0.90)	(3.63, 1.66)	(5.27, 2.41)
0.20	(2.97, 0.80)	(3.37, 0.90)	(3.93, 1.65)	(5.55, 2.32)
$$n=20$$				
0.03	(2.56, 0.40)	(2.70, 0.56)	(2.77, 1.55)	(4.17, 2.21)
0.05	(2.75, 0.55)	(2.70, 1.35)	(3.27, 1.91)	(4.65, 2.71)
0.10	(3.08, 0.60)	(3.10, 1.83)	(3.84, 2.27)	(5.85, 3.06)
0.20	(3.07, 0.79)	(3.43, 1.69)	(4.20, 2.08)	(5.93, 2.93)

$\omega$	$ARL_0=500$			
$$n=5$$				
0.03	(2.145, 0.534)	(2.48, 0.67)	(2.67, 0.67)	(3.86, 2.47)
0.05	(2.30, 0.40)	(2.63, 0.98)	(2.99, 0.83)	(4.31, 1.67)
$$n=10$$				
0.03	(2.51, 0.53)	(2.67, 0.68)	(2.89, 1.08)	(4.39, 1.61)
0.05	(2.75, 0.54)	(2.78, 0.96)	(3.28, 1.41)	(4.48, 2.96)
0.10	(2.85, 0.57)	(2.99, 1.95)	(3.69, 1.44)	(5.09, 3.59)
0.20	(2.87, 0.68)	(3.15, 1.26)	(3.82, 2.22)	(5.38, 2.18)
$$n=15$$				
0.03	(2.50, 0.47)	(2.50, 0.92)	(2.99, 1.58)	(4.29, 1.93)
0.05	(2.76, 0.54)	(2.96, 0.91)	(3.27, 1.91)	(4.79, 2.90)
0.10	(2.99, 0.91)	(3.19, 0.99)	(3.73, 1.97)	(5.28, 2.78)
0.20	(2.97, 1.21)	(3.41, 1.05)	(4.02, 2.36)	(5.65, 3.39)
$$n=20$$				
0.03	(2.88,0.39)	(2.75, 0.66)	(3.17, 1.12)	(4.38, 1.62)
0.05	(2.75, 0.78)	(2.84, 1.07)	(3.46, 1.37)	(4.98, 1.85)
0.10	(3.09, 0.80)	(3.40, 1.55)	(4.24, 1.90)	(5.98, 2.70)
0.20	(3.17, 0.83)	(3.55, 1.28)	(4.22, 1.67)	(5.96, 2.79)

## Performance evaluation of proposed charts

Generally, the average run length (ARL) is used to determine the proposed control charts’ performance. The ARL of the control charts, when the process is in control, is denoted by $ARL_0$, and when the process is out of control is denoted by $ARL_1$. The comparative performance of proposed charts is made under different symmetric distributions and sampling schemes based on out-of-control values of run-length characteristics ($ARL_1, MDRL_1,$ and $SDRL_1$) to identify a shift. Moreover, the specified $ARL_0$ value, a control chart with smaller $ARL_1$ is considered more efficient in detecting the shift. The shift in the process location is presented by $\delta$ where $\delta =0$, indicates the process in-control and $\delta > 0$ indicates the out-of-control shift in the process. Various methods are utilized for calculating the ARLs of the EWMA control chart, such as Markov Chain, Monte Carlo Simulation, and integral equation. In this study, the ARL of the proposed charts is calculated through Monte Carlo Simulation using the R programming language with different values of the design parameters due to the flexibility and accuracy to switch different conditions as competed to other methods. After selecting the fixed in-control ARL for the proposed chart with designed parameters, the RL characteristics like ARLs, SDRLs, and MDRLs are calculated using the following algorithm.

**Table:** 

Algorithm for calculating the proposed charts run length characteristics	
1. Create 1000 random samples with bivariate normal distribution, given by $(X, Y)\sim N_2 (\theta _{X_0}+\delta \sigma _{X},\mu _Y=\theta _0, \sigma _X=1, \sigma _Y=1, \rho )$. Initially, if $\delta =0$ the process shows IC.	
2. Calculate the EWMA-SR, HWMA-SR, and Auxiliary HWMA-SR statistics ($Z_j$), ($H_j$), and ($M_j$) for each random sample using Eqs. (2,4), and (10).	
3. Calculate the variance of all plotting statistics using Eqs. (3, 5), and (11), then calculate both chart’s control limits using randomly selected values of control limits coefficients ($k_1,k_2$) for a specified in-control ARL.	
4. Calculate the control limits and equate the plotting statistics with their respective control limits until the first OOC run length is noted.	
5. Step 1 to 4 is repeated for *N* time (say $N=10,000$) to obtain an IC RL in every case.	
6. Calculate the mean, median, and standard deviation of the *RLs* obtained in step 5 to get the ARL, MDRL, and SDRL as follows:	
$$\begin{array}{c} ARL= mean(RL)\\ MDRL=median(RL)\\ SDRL=SD(RL) \end{array}$$	
7. If the calculated ARL is equivalent to the specified ARL, formerly go to the next step by preserving the recorded values of control charts constants. Otherwise, repeat steps 1 to 6 in order to get the fixed in-control ARL.	
8. Generate the subgroups sample size from normal distribution by introducing a shift $(\delta \ne 0)$ in the process location (out of control process). For example, the sample can be drawn from the normal distribution with mean ($\theta _1=\theta _0+\delta \sigma _0$) and standard deviation $\sigma _0=1$.	
9. Repeat steps 2, 4, and 6 for N times under the shifted process and calculate the out-of-control $ARL_1, MDRL_1$, and $SDRL_1$.	

### Run length distribution of the nonparametric proposed repetitive charts

Usually, the run-length distribution is used for the assessment of control chart performance. The most famous methods to assess the performance of the run-length distribution are average run length (ARL), median run length (MDRL), and standard run length (SDRL). These methods are used to check the control chart’s ability to identify the exact value of the $\delta$ in the process parameters. The control chart with smaller values of $ARL_1$ for a fixed size of the shift is measured as an effectual chart. In this paper, we have used the fixed or IC $ARL_0=500$ for the calculation of the RL distribution in the standard normal distribution, *N*(0, 1), and heavy-tailed symmetric distributions, which include the logistic distribution, LG($0,\sqrt{3}/\pi$), the student’s t distribution with 4 and 8 degrees of freedom, Laplace($0,1/\sqrt{2}$), see detain in Table 4.

**Table 4:** PDFs of the Distributions used in this manuscript.

(1) Normal Distribution $f(X)=\frac{1}{\sigma \sqrt{2\pi }}e^{-\frac{1}{2}(\frac{x-\mu }{\sigma })^2}$, where $\mu =0$ and $\sigma =1$	
R function for Normal Distribution: rnorm(n, $\mu$, sd)	
(2) Students $t(v) f(X)=\frac{\Gamma (\frac{v+1}{2})}{\Gamma (\frac{v}{2})\sqrt{v\pi }}(1+\frac{X^2}{v})^{(\frac{v+1}{2})}$, where; $\mu =0$ and $\sigma ^2=\frac{v}{v-2}$ and $v=4,8$ are taken.	
R function for student distribution: rt(n, $\mu$, sd)	
(3) Logistic $f(X)=\frac{e^{-\frac{\pi X}{\sqrt{3}}}}{\frac{\sqrt{3}}{\pi }(1+e^{\frac{-\pi X}{\sqrt{3}}})^2}$,where $\mu =0$ and $\sigma ^2=\frac{3}{\pi ^2}$	
R function for logistic distribution: rlogis(n, $\mu , \sqrt{(}3)/\pi$)	
(4) Laplace $f(X)=\frac{1}{2}e^{-|X|}$, where $\mu =0$ and $\sigma ^2=\frac{1}{2}$	
R function for Laplace distribution: rlapce(n, $\mu$, sd)	

**Figure 2:**
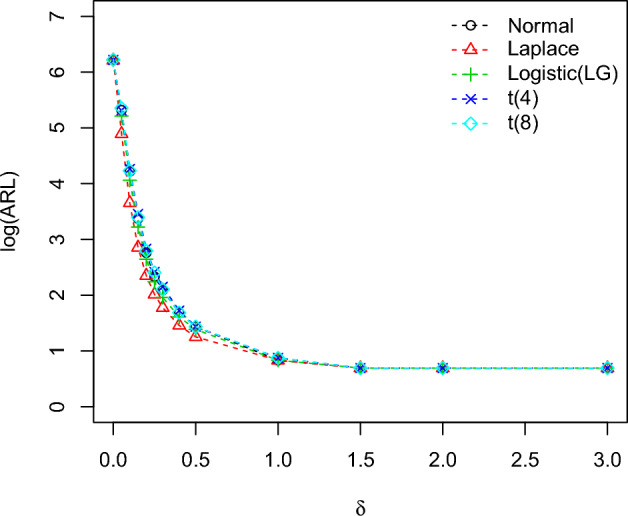
The ARLs comparison of EWMA-SR repetitive chart for various shifts with designed parameters $n=10$,$\omega =0.10$ for different distributions.

**Figure 3:**
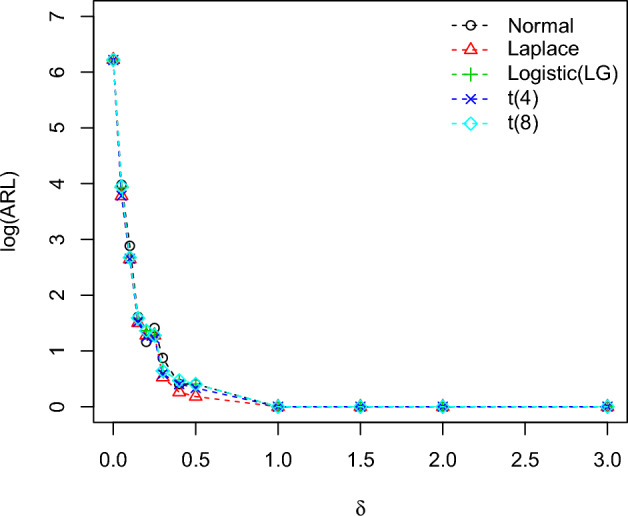
The ARLs comparison of HWMA-SR repetitive chart for various shifts with designed parameters $n=10, \omega =0.10$ for different distributions.

**Figure 4:**
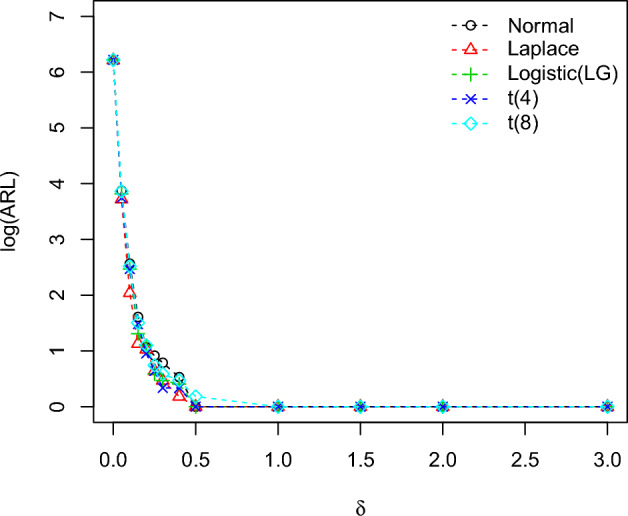
The ARLs comparison of Auxiliary HWMA-SR repetitive charts for various shifts with designed parameters $n=10, \omega =\rho =0.10$ for different distributions.

To calculate the OOC run-length distributions $ARL_1$ in the process is familiarized in terms of the method’s standard deviation, i.e.,$\theta _1=\theta _0+\delta \sigma$, where $\delta$ calculates the scale of shift. The values of the plotting charts constants ($k_1,k_2$) with the selected set of designed parameters are taken from Tables 2 and 3 and results of all the distribution *ARL* are mentioned in Table 5. From Table 5, the IC ARL values of the planned charts remain similar for all distributions which is 500, which is mentioned with the nonparametric control chart scheme. So the charting characteristics OOC values ($ARL_1$) are perfumed satisfactory and decline quickly with the rise of the process shift irrespective of the type of the distributions. The comparison between the *ARL* values for all the distributions of the planned control charts is revealed in Figs. 2, 3, 4 for identified values of IC $ARL_0=500$. These Figures show that the proposed charts performed well compared to other distributions when they follow the Laplace distribution because the OOC $ARL_1$ of the values of the Laplace distribution decline quickly with the rise of the shift process as compared to the other distributions.

**Table 5:** The run length characteristic (ARL, SDRL (in 2nd row) and MDRL (in 3rd row)) of the proposed EWMA-SR, HWMA-SR, and Auxiliary HWMA-SR repetitive charts with $n=10, \omega =\rho =0.10$ for different distributions.

Charts	shift												
	0.00	0.05	0.10	0.15	0.20	0.25	0.30	0.40	0.50	1.0	1.5	2.0	3.0
Normal (0, 1)													
EWMA-SR Repetitive	500.1	204.0	67.9	29.2	15.8	10.3	8.1	5.4	4.2	2.3	2	2	2
	503.7	207.7	60.5	23.7	10.6	6.5	4.3	2.2	1.4	0.5	0.1	0	0
	365	140	51	22	13	9	7	5	4	2	2	2	2
HWMA-SR Repetitive	500.3	53.5	17.9	5.0	3.2	4.1	2.4	1.5	1.5	1	1	1	1
	774.8	42.7	12.6	4.3	2.5	2.7	2.2	1.9	1.8	0	0	0	0
	132.5	53	16	3	2.2	4	1	1	1	1	1	1	1
AHWMA-SR Repetitive	501.7	48.1	13	5	2.9	2.5	2.2	1.7	1	1	1	1	1
	506.2	35.3	12.8	3.6	1.6	1.5	1.3	1.1	0	0	0	0	0
	275	38.5	8	3.5	2.6	1.5	1	1	1	1	1	1	1
$$\text{Laplace}\,(0,1/\sqrt{2})$$													
EWMA-SR Repetitive	499.0	133.5	38.7	17.4	10.5	7.5	5.9	4.3	3.5	2.3	2	2	2
	488.7	120.4	31.2	12.0	6.4	3.9	2.7	1.6	1.1	0.4	0.2	0	0
	355.0	95.5	30.0	14.0	9.0	6.5	5.0	4.0	3.0	2	2	2	2
HWMA-SR Repetitive	504.5	43.8	14.1	4.5	3.6	3.6	1.7	1.3	1.2	1	1	1	1
	564.6	43.4	8.6	4.2	2.8	3.4	0.9	0.7	0	0	0	0	0
	291.5	35	12	4	3.5	3	1	1	1	1	1	1	1
AHWMA-SR Repetitive	499.4	41.4	7.7	3.1	2.8	1.9	1.6	1.2	1	1	1	1	1
	632.1	67.9	9.6	2.7	2.5	1.2	1	0.6	0	0	0	0	0
	261.5	15	3.5	3	2	1	1	1	1	1	1	1	1
$$\text{LG}\,(0,\sqrt{3}/\pi )$$													
EWMA-SR Repetitive	502.7	183.3	58.1	25.1	14.1	9.4	7.1	5.0	4	2.3	2	2	2
	476.1	188.7	51.8	20.1	9.3	5.2	3.6	1.9	1.3	0.5	0.2	0	0
	381.0	130.0	43.5	19.0	12.0	8.0	6.0	5	4	2	2	2	2
HWMA-SR Repetitive	500.6	50.9	14.5	4.9	3.9	3.6	1.9	1.6	1.5	1	1	1	1
	383.1	57.5	16.2	2.9	4.6	1.6	1.3	0.9	0	0	0	0	0
	357.5	25.5	14	3	3	3.5	1	1	1	1	1	1	1
AHWMA-SR Repetitive	502.8	45.5	11.8	3.7	3	1.8	1.6	1.5	1	1	1	1	1
	545.4	41.6	9.6	3.5	3	1.7	1.6	0.9	0	0	0	0	0
	369.5	38	10	2.5	2	1.3	1.5	1	1	1	1	1	1
Student’s t-Distribution (4)													
EWMA-SR Repetitive	501.6	203.4	71.0	31.7	17.0	11.2	8.6	5.6	4.2	2.4	2	2	2
	496.4	204.3	63.7	26.7	12.4	7.1	4.9	2.5	0.5	0.5	0.1	0	0
	351.5	141.0	53.0	23.0	13.0	9.0	7.0	5.0	2	2	2	2	2
HWMA-SR Repetitive	501.0	44.0	14.2	4.6	3.5	3.4	1.8	1.5	1.4	1	1	1	1
	438.2	60.5	21.6	2.8	2.5	2.2	1.4	0.6	0.5	0	0	0	0
	327.5	32.5	4.5	4	3	3	1	1	1	1	1	1	1
AHWMA-SR Repetitive	502.1	42.3	11.8	4.4	2.6	1.9	1.4	1.4	1	1	1	1	1
	438.9	38.5	12.4	3.2	3.2	1.1	0.9	0.7	0	0	0	0	0
	436.5	23	4	3.5	1	1	1	1	1	1	1	1	1
Student’s t-Distribution (8)													
EWMA-SR Repetitive	503.8	211.4	68.8	29.8	16.4	11.1	8.2	5.4	4.2	2.4	2	2	2
	503.9	204.1	62.4	24.3	11.1	6.8	4.3	2.3	1.5	0.5	0.2	0	0
	342	158	50	22	13	9	7	5	4	2	2	2	2
HWMA-SR Repetitive	503.2	51.9	14.5	4.9	3.9	3.6	1.9	1.6	1.5	1	1	1	1
	580.9	46.5	9.8	4.4	4.6	2.8	1.3	1.2	1.1	0	0	0	0
	190	45	14.5	4	3	3	1.5	1	1	1	1	1	
AHWMA-SR Repetitive	502.1	47.6	12.4	4.5	3	2.1	1.8	1.6	1.2	1	1	1	1
	416.9	36.1	13.4	4.7	2.3	1.8	1.5	1	0.3	0	0	0	0
	387	40.5	6.5	2.5	1.8	1.5	1	1	1	1	1	1	1

### Results and discussion

The algorithm mentioned in the Section "The design of all proposed nonparametric signed-rank repetitive control charts" with different distributions has been used to acquire the desired ARLs, SDRLs, and MDRLs values for IC and for the shifted procedure for $n=15, 20, \omega =0.03, 0.05$, and 0.20, and $\rho =0.25, 0.5,$ and 0.75. Different pairs of coefficient constants result in $ARL=500$ for normal distribution but only one of those which gives the smallest values of OOC ARL is selected for the simulation study. So, the values for these constants calculated for the proposed charts are revealed in Tables 1 and 2 for different values of $\omega , \rho$, and sample size (n). Values of run-length characteristics (ARLs, SDRLs in 2nd row, and MDRLs in 3rd row) are mentioned in Tables 8 and 9. Overviewing Tables 5, 6, 7, 8, 9, the analysis uncovers the following points. For Auxiliary HWMA-SR repetitive chart from Table 8:

1. It is noticed that for the specified shift ($\delta$) values and $\rho$ values, the proposed chart is performed efficiently for small values of smoothing parameter ($\omega$) as compared to large values. For example, when $\delta =0.1, \rho =0.25$, and $\omega =0.03, 0.05, 0.2$ the values of $ARL_1$ are 23.4, 29.3, and 31.9. So, the chart detects the shift in the process median earlier as $\omega$ has smaller values.
2. For fixed $\omega$ and $\delta$ values, the chart is performed well when $\rho$ value is large. For example, when $\omega =0.05, \delta =0.1$, and $\rho =0.25, 0.5$, and 0.75, $ARL_1$ values are 29.3, 24.9, 21.2, respectively. So, the increase in the link configuration between the process variable and the Auxiliary variable leads to rising the detection capability of the chart at all levels of shift.
3. As shift increases, the $ARL_1, MDRL_1$, and $SDRL_1$ values contacted to 1 and 0, separately, particularly for large $\rho$ values; mean, the charts reveal large shifts quickly.
4. As likely, the in-control RL is confidently skewed from the time when $ARL_0 > MDRL_0(P_{50})$. For instant, when $\rho =0.25$,$\omega =0.03, \delta =0$, the values of MDRL=429.5 that is less than to $ARL_{0}=499.3$.

For EWMA-SR and HWMA-SR repetitive charts from Table 9:

1. The $ARL_1$ values increases as the values of smoothing constant ($\omega$) increase at a fixed shift. For example, when $\omega =0.03, 0.05, 0.20$, and $\delta =0.1$, the $ARL_1$ values for EWMA-SR repetitive are 23.5, 36.8, and 53.6 while, the $ARL_1$ values for HWMA-SR repetitive chart are 17.2, 21.2, and 23.1.
2. $ARL_1$ is large for small shifts and decreases when the shift increases. For example when $\omega 0.05, \delta =0.1, 0.5, 1.0, 1.5, 2,$ and 3, the $ARL_1$ for EWMA-SR repetitive chart are 36.8, 4.4, 2, 2, 2, and 2, while as the $ARL_1$ for HWMA-SR repetitive chart are 21.2, 1.7, 1, 1, 1, and 1.
3. From Tables 5, 6, 7, 8, 9, it is noted that the proposed charts performed well for the Laplace distribution as compared to other distributions. Moreover, the Auxiliary HWMA-SR repetitive chart is more efficient to detect the shift in the process as compared to the proposed EWMA-SR repetitive and HWMA-SR repetitive charts.

### Comparison with the existing control charts

In this section, comparisons of the proposed charts with their counterparts are made. The run-length distribution results are generated by using the Monte Carlo simulation. The detailed comparison of the nonparametric signed-rank based repetitive sampling proposed charts: EWMA-SR repetitive, HWMA-SR repetitive, and AHWMA-SR repetitive, with some existing nonparametric signed-rank based control charts: EWMA-SR proposed by Graham et al.^19^, HWMA-SR chart proposed by Raza et al.^26^ and the auxiliary-based HWMA-SR chart in terms of the average run length values. These results are determined so that the IC ARL remains 500 not only for normal distribution but also determined for Laplace, logistic, and t-distribution with 4 and 8 degrees of freedom in Table 8. For comparison of the proposed and existing charts with a single sampling scheme and repetitive sampling scheme, we used the sample size $n=10, \delta =0.25, 0.5, 1, 2, 3$, and $\omega =\rho =0.05$. For comparison with the auxiliary-based chart, we used different values of $\rho$, i.e., $\rho =(0.05, 0.25, 0.5, 0.75)$. In all situations, the designed parameters were set to values that specific $ARL_0= 500$. The results displayed that optimized $\delta$ at $\omega =(0.03, 0.05, 0.10,$ and 0.20).

**Table 6:** ARL comparison from different distributions of EWMA-SR^19^, HWMA-SR^26^, and AHWMA-SR control chart under classic statistics (CS) versus Repetitive Sampling(RS) when $n=10, \rho =\omega =0.05$ and $ARL_0=500$.

Charts	Scheme	$\delta$							
		0.05	0.10	0.15	0.25	0.5	1.0	2.0	3.0
Normal (0,1)									
EWMA-SR	Repetitive sampling	168.2	55.3	25.9	11.2	4.9	3.1	3	3
	Under CS^19^	179.1	65.4	35.9	16.8	7.6	4.5	4	4
HWMA-SR	Repetitive sampling	81.1	17.9	10.6	4.1	1.5	1	1	1
	Under CS^26^	134.9	55.1	30.6	11.8	4.4	2.8	1	1
Auxiliary HWMA-SR	Repetitive sampling	67.9	13.7	9.1	2.6	1	1	1	1
	Under CS	125.8	56.9	27.1	10.6	4.8	1.9	1	1
Laplace (0,1/$\sqrt{2}$)									
EWMA-SR	Repetitive sampling	112.8	32.5	15.9	8.3	4.3	3.0	3	3
	Under CS^19^	116.7	42.6	22.6	12.7	6.5	4.3	4	4
HWMA-SR	Repetitive sampling	61.1	13.6	7.5	5.2	1	1	1	1
	Under CS^26^	95.4	32.4	17.8	8.2	3.6	1.8	1.1	1
Auxiliary HWMA-SR	Repetitive sampling	53.2	14.4	5.8	2.4	1	1	1	1
	Under CS	90.6	28.9	15.3	6.1	3.4	1.8	1	1
LG (0,$\sqrt{3}/\pi$)									
EWMA-SR	Repetitive sampling	144.9	43.5	21.6	10.1	4.7	3.1	3	3
	Under CS^19^	153.4	55.8	30.4	15.3	7.2	4.4	4	4
HWMA-SR	Repetitive sampling	69.3	16.8	8.8	1.4	1	1	1	1
	Under CS^26^	101.7	47.9	20.8	10.4	4.0	1.9	1	1
Auxiliary HWMA-SR	Repetitive sampling	55.6	15.3	7.8	1.8	1.1	1	1	1
	Under CS	98.1	50.5	18.8	9.6	3.5	1.7	1	1
Student’s t-Distribution (4)									
EWMA-SR	Repetitive sampling	164.9	51.5	24.7	11.5	5.2	3.1	3	3
	Under CS^19^	176.4	60.5	34.7	13.1	6.5	4.3	4	4
HWMA-SR	Repetitive sampling	73.5	12.0	6.4	1.5	1.4	1	1	1
	Under CS^26^	124.6	49.8	21.6	8.7	3.5	1.8	1.1	1
Auxiliary HWMA-SR	Repetitive sampling	56.3	9.3	6.2	3.5	1.7	1	1	1
	Under CS	86.9	32.1	20.6	9.8	4.3	2.4	1	1
Student’s t-Distribution (8)									
EWMA-SR	Repetitive sampling	166.2	54.7	25.3	11.5	5.2	3.1	3	3
	Under CS^19^	178.6	63.2	35.7	16.6	7.8	4.5	4	4
HWMA-SR	Repetitive sampling	80.4	16.2	9.3	2.0	1.3	1	1	1
	Under CS^26^	128.5	54.2	25.8	12.5	4.5	2.2	1.1	1
Auxiliary HWMA-SR	Repetitive sampling	60.3	12.4	7.3	3.9	1.2	1	1	1
	Under CS	109.5	47.4	22.1	9.6	4.4	1.8	1	1

**Table 7:** The OOC RL characteristics values (the first row contains ARL, 2nd row contain SDRL, and 3rd row contains MDRL values) of the EWMA-SR(E-SR), HWMA-SR(H-RS), Auxiliary HWMA-SR, and proposed repetitive charts (EWMA-SR*, HWMA-SR*, and Auxiliary HWMA-SR) for, n=10, $ARL_0= 500$ and different values of shift plus designed parameters.

$\omega$	$\delta$	Classical Charts				Auxiliary HWMA-SR				Auxiliary HWMA-SR Repetitive			
						$\rho$				$\rho$			
		^19^	^26^	E-SR	H-SR	0.05	0.25	0.5	0.75	0.05	0.25	0.5	0.75
0.03	0.1	59.6	44.8	29.5	13.2	36.4	33.9	27.7	25.3	11.2	10.9	9.8	9.7
		40.3	29.5	22.3	11.6	18	29.6	21.6	23.6	10.8	18.5	7.6	9.1
		49	44	23	11.5	27	30	18.5	13	9	4	7.5	7
	0.5	8.5	3.7	3.7	1.3	3.1	2.9	2.6	2.1	1.2	1.2	1.1	1.1
		1.9	1.8	1.2	0.6	1.9	1.4	1.6	1.7	0.6	0.6	0.8	0.8
		8	3	4	1	3	2.8	2.5	1	1	1	1	1
	1.0	5.2	1.5	2.2	1	1.6	1.5	1.2	1	1	1	1	1
		0.5	0.8	0.4	0	0.9	0.7	0.6	0	0	0	0	0
		5	1	2	1	1	1	1	1	1	1	1	1
	3.0	4	1	2	1	1	1	1	1	1	1	1	1
		0	0	0	0	0	0	0	0	0	0	0	0
		4	1	2	1	1	1	1	1	1	1	1	1
0.05	0.1	64.1	48.5	50.1	15.4	45	40.2	38.7	36.7	12.9	11.8	11.6	10.9
		48.5	30.7	37.9	18.4	41.7	29.1	31.6	48.7	9.9	11.2	9.6	10.2
		49	45	39	10	38.5	36.5	23	15	11	7.5	9	9
	0.5	7.6	4.1	5	1.6	4.8	4.3	3.3	3.2	1.8	1.5	1.4	1.3
		1.9	1.9	1.4	1.3	1.8	2.3	1.5	1.3	1.4	0.8	0.6	0.8
		5	4	5	1	4.5	4.2	3	3	1	1	1	1
	1.0	4.4	1.8	3.1	1.5	2.1	1.9	1.8	1.5	1	1	1	1
		0.6	0.9	0.4	0.3	0.9	0.9	1.0	0.8	0	0	0	0
		4	1	3	1	2	1.5	1	1	1	1	1	1
	3.0	4	1	2	1	1	1	1	1	1	1	1	1
		0	0	0	0	0	0	0	0	0	0	0	0
		4	1	2	1	1	1	1	1	1	1	1	1
0.10	0.1	78.2	52.1	64.8	17.9	52.1	44.5	39.5	36.3	13.0	12.7	12.2	11.5
		65.0	32.9	56.9	12.6	48.2	28.0	25.4	30.5	12.8	12.4	12.7	9.9
		60.0	47.5	47.0	16.0	39.0	40.0	30.5	26.0	8.0	8.5	7.5	10.5
	0.5	6.6	4.8	4.2	1.8	5.0	4.1	3.6	3.5	2.0	1.6	1.5	1.3
		2.1	2.1	1.4	0.8	2.8	1.3	1.8	1.6	1.7	1.0	1.0	0.6
		6	4	4	1	4.5	3.5	3.5	3.5	1.0	1.0	1.0	1.0
	1.0	4.1	2.1	2.3	1.0	2.7	1.9	1.7	1.4	1.0	1.0	1.0	1.0
		0.5	0.9	0.5	0	0.6	0.9	0.8	0.6	0	0	0	0
		4.0	2.0	2.0	1.0	2.0	1.5	1.5	1.0	1.0	1.0	1.0	1.0
	3.0	3	1	2	1	1	1	1	1	1	1	1	1
		0	0	0	0	0	0	0	0	0	0	0	0
		3	1	2	1	1	1	1	1	1	1	1	1
0.2	0.1	116.1	52.9	92.1	21.3	70.5	53.1	44.7	40.5	20.7	19.3	17.2	15.1
		111.6	35.3	86.7	15.2	46.3	50.1	18.1	37.1	16.2	14.8	12.8	16.9
		82.0	46.5	63.5	17.5	58.5	37.5	43.5	33.5	17.0	14.0	15.0	9.5
	0.5	5.8	4.7	3.1	1.2	6.1	5.2	5.0	4.8	2.3	2.0	1.6	1.2
		3.0	1.9	1.2	0.4	3.2	2.1	1.8	2.2	1.3	1.9	0.8	0.6
		5.0	4.0	3.0	1.0	5.5	5.0	5.0	4.5	2.0	1.0	1.0	1.0
	1.0	3.1	2.2	1.7	1.0	2.7	2.0	2.0	2.0	1.1	1.1	1.1	1.0
		0.5	0.7	0.5	0	0.5	0.4	0.4	0.3	0.3	0.3	0.3	0
		3.0	2.0	2.0	1.0	2.0	2.0	2.0	2.0	1	1	1	1
	3.0	2	1	1	1	1	1	1	1	1	1	1	1
		0	0	0	0	0	0	0	0	0	0	0	0
		1	1	1	1	1	1	1	1	1	1	1	1

**Figure 5:**
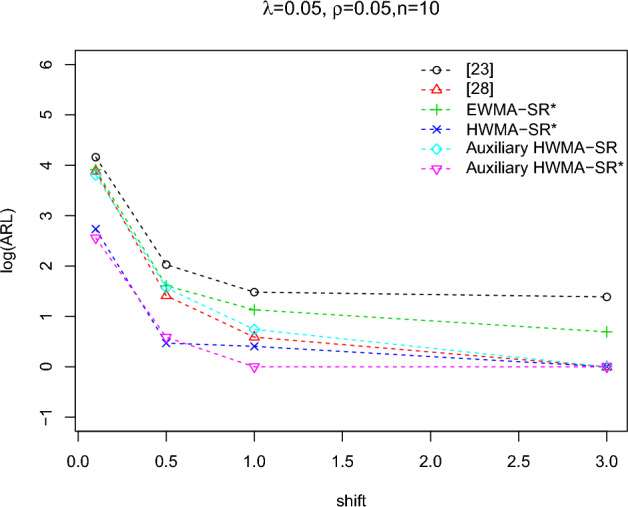
The ARLs-based comparison presentation of proposed charts and existing charts.

The comparison results are mentioned in Tables 6, 7 and Fig. 5. From Table 6, the comparative analysis uncovers the following points:

1. The repetitive sampling scheme detects a shift earlier as compared to the single sampling scheme.
2. The charts perform well as compared to other distributions when they follow the Laplace distribution.
3. For example, the EWMA-SR with repetitive sampling scheme performs the best as compared to a single sampling scheme, it is noticed that, at $\delta =0.25, \omega =0.05$, and $n=10$, the $ARL_1$ values are 11.2, 8.3,10.1, and 11.5 for repetitive sampling with different distributions and the $ARL_1$ values are 16.8, 12.7, 15.3, and 13.1 for single sampling scheme with different distributions.
4. When we check the performance of both schemes for the HWMA-SR chart, we also noticed that the RS scheme performs the best as compared to the SS scheme. For example, at $\delta =0.5,\omega =0.05$, and $n=10$, the $ARL_1$ values for the RS scheme are 1.5,1,1,1.4 and the $ARL_1$ values for SS scheme are 11.8,8.2,10.4,8.7.
5. As when we try to check the performance of both schemes for the auxiliary HWMA-SR chart, the RS scheme performance is also best as compared to the SS scheme (see cf. Table 6).

From Table 7, the comparative analysis uncovers the following points:

1. The proposed charts detect shifts earlier as compared to counterpart charts.
2. The $ARL_1$ values are increased for all charts when smoothing parameters are increasing at all levels of shift.
3. As shown in Table 7 and Fig. 5, the proposed Auxiliary HWMA-SR repetitive chart outperformed the classical charts in detecting the shift at all levels, especially when $\rho >0.05$.
4. ***Proposed EWMA-SR repetitive Vs Existing EWMA-SR charts.*** The run-length distribution values for the proposed and existing chart are plotted in Table 9. The comparison uncovers that EWMA-SR repetitive chart performs the best at the different levels of shifts plus the different levels of smoothing parameter ($\omega$) with $n=10$. For example, when $\omega =0.03, n=10$, and $\delta =0.10,0.5,1.0,2.0, and 3.0$, the $ARL_1$ values for the proposed chart are 29.5, 3.7, 2.2, 2, 2, and 2, whereas the $ARL_1$ for the existing chart are 59.6, 8.5, 5.2, 4, 4, and 4 (cf. Table 7). From Table 7 results, it is observed that with all selections of designed parameters, the proposed chart performs more efficiently as compared to the existing chart. Moreover, these results show that the proposed chart is better in terms of detection ability at all levels of shifts than the existing chart.
5. ***Proposed HWMA-SR repetitive Vs Existing HWMA-SR charts.*** The proposed chart and existing chart $ARL_1$ values are compared in Table 9 at a different level of shifts. So, the results have shown that the proposed chart has significantly better performance as compared to the counterpart chart. For instant, with $n=10, \omega =(0.03,0.05,0.1$, and 0.2), and $\delta =0.1$, the proposed chart $ARL_1=13.2, 15.4, 17.9, 21.3$ whereas the corresponding $ARL_1=44.8, 48.5, 52.1, 52.9$ for HWMA-SR chart. From these results, we noticed a considerably improved performance of the HWMA-SR repetitive chart as compared to the HWMA-SR chart. Moreover, we also noticed that when $\omega$ increases the $ARL_1$ also increases at the same level of shift and sample size. The same trend was also noticed in all other charts.
6. ***Proposed Auxiliary HWMA-SR repetitive Vs Auxiliary HWMA-SR charts.*** The run-length acatalectics values of the proposed chart are reported in Table 7 and it is observed that the $ARL_1$ values of the proposed chart are less than the Auxiliary HWMA-SR chart, under all shifts in the process. Moreover, we also have seen the decreasing trend at all levels of the shift in the $ARL_1$ values when $\rho$ values increase and $ARL_1$ values increased when $\omega$ values increase. For example, when $n=10, \omega =0.10, 0.1$ and $\rho =(0.05, 0.25, 0.5, 0.75)$, the $ARL_1$ values for Auxiliary HWMA-SR repetitive chart are 13, 12.7, 12.5, and 11.5 against the $ARL_1$ values for the existing chart are 52.1, 44.5, 39.6, and 36.3. Furthermore, when $n=10, \rho =0.50$, and $\omega =(0.03, 0.05, 0.1, 0.2)$, the $ARL_1$ values for the proposed chart are 9.8, 11.6, 12.2, and 17.2 while the $ARL_1$ values for the existing chart are 27.7, 38.7, 39.5, and 44.7. So, all the results clearly indicate the superiority of the Auxiliary HWMA-SR repetitive chart against the Auxiliary HWMA-SR char at all levels of shifts (cf. Table 7 and Fig. 5).

**Table 8:** The run length characteristic (ARL, SDRL (in 2nd row) and MDRL (in 3rd row)) of the proposed Auxiliary HWMA-SR repetitive chart with $n=15$, $ARL_0=500$, different values of $\omega$, and different values of $\rho$ for different distributions.

$\delta$	$\rho =0.25$			$\rho =0.5$			$\rho =0.75$		
	$\omega =0.03$	$\omega =0.05$	$\omega =0.2$	$\omega =0.03$	$\omega =0.05$	$\omega =0.2$	$\omega =0.03$	$\omega =0.05$	$\omega =0.2$
Normal (0,1)									
0.0	499.3	501.5	502.9	500.2	499.0	499.1	502.2	501.2	501.9
	452.4	439.8	387.7	566.2	268.7	578.5	503.7	447.8	581.9
	429.5	423	400.5	321.5	441	329	359.5	359.5	330.5
0.1	23.4	29.3	31.9	20.6	24.9	27.2	19.8	21.2	25.7
	15.9	20.9	39.1	12.1	21.5	19.5	13.4	13.4	30.3
	21	23	8	17	16.5	25.5	15.5	19	10
0.5	1.7	1.9	1.9	1.5	1.9	2	1.3	1.6	2
	0.9	1.1	0.9	1.1	1.3	0.8	0.9	0.8	1.3
	1	1	1	1	1	2	1	1	1
1	1	1	1	1	1	1	1	1	1
	0	0	0	0	0	0	0	0	0
	1	1	1	1	1	1	1	1	1
3	1	1	1	1	1	1	1	1	1
	0	0	0	0	0	0	0	0	0
	1	1	1	1	1	1	1	1	1
Laplace (0,$1/\sqrt{2}$)									
0.0	502.4	500.8	501	501.9	499.7	503.4	500.4	499.6	502.4
	405.3	331.5	324.3	372.3	399.9	346.8	413.4	322.2	323.3
	439.5	499	457.5	481	445.5	496.5	422	415	478.5
0.1	15.3	16.4	19.1	13.8	14.8	18.5	11.7	12.4	17.3
	15.1	17.8	17.1	15.2	11.5	25.5	9.5	13.9	12.9
	10.5	7	14	6	14	10	8	5.5	16
0.5	1.4	1.5	1.6	1.2	1.4	1.6	1.2	1.3	1.5
	0.8	1.1	0.8	0.6	0.8	0.9	0.6	0.6	0.8
	1	1	1	1	1	1	1	1	1
1	1	1	1	1	1	1	1	1	1
	0	0	0	0	0	0	0	0	0
	1	1	1	1	1	1	1	1	1
3	1	1	1	1	1	1	1	1	1
	0	0	0	0	0	0	0	0	0
	1	1	1	1	1	1	1	1	1
LG (0,$\sqrt{3}/\pi$)									
0.0	501.8	504.3	503.2	506.6	499.1	500.7	500.2	499.8	500.5
	368.3	418.8	439.2	530.8	402.7	404.9	429.7	291.4	474.6
	452	415.5	490	358	434	380.5	441.5	487	254
0.1	17.5	21.3	29.9	16.1	19.4	25.8	13.7	18.1	24.4
	16.9	27.7	45.1	21.7	17.9	15.9	15.9	16.3	15.2
	15.5	11.5	12.5	5.5	16	21.5	7	12	23.5
0.5	1.5	1.7	1.9	1.4	1.6	1.8	1.3	1.4	1.7
	1.1	1.1	0.9	0.9	0.9	1.0	0.9	0.8	0.9
	1	1.5	1	1	1	1	1	1	1.5
1	1	1	1	1	1	1	1	1	1
	0	0	0	0	0	0	0	0	0
	1	1	1	1	1	1	1	1	1
3	1	1	1	1	1	1	1	1	1
	0	0	0	0	0	0	0	0	0
	1	1	1	1	1	1	1	1	1
Student’s t-Distribution (4)									
0.0	505.9	503	499.1	501.8	503.2	502	503.7	503.6	501.3
	353.9	324.1	384.2	485.6	359.6	387.8	490.4	503.7	502.3
	492.5	479.5	468	369.5	486	459.5	352.2	422.5	456.1
0.1	20.2	21.8	22.5	17.4	20.6	21.1	15.3	19.1	20.2
	17.4	20.7	36.0	13.1	17.8	13.3	16.2	20.6	14.2
	15	16.5	7.5	14	14	20	8.5	11	14
0.5	1.5	1.6	1.7	1.4	1.5	1.6	1.3	1.4	1.5
	1.1	1.2	0.8	0.8	1.1	1.1	0.6	0.8	0.8
	1	1	1.5	1	1	1	1	1	1
1	1	1	1	1	1	1	1	1	1
	0	0	0	0	0	0	0	0	0
	1	1	1	1	1	1	1	1	1
3	1	1	1	1	1	1	1	1	1
	0	0	0	0	0	0	0	0	0
	1	1	1	1	1	1	1	1	1
Student’s t-Distribution (8)									
0.0	505.5	500.0	503.7	499.7	498.0	501.9	507.2	502.0	502.8
	284.1	310.4	350.8	384.6	413.1	521.1	335.7	162.3	271.5
	490.5	456.5	447.5	493.0	401.5	394.0	459.5	425.0	435.0
0.1	21.3	28.2	29	19.1	25.4	26.8	18	23.5	24.9
	23.5	30.5	34.4	17.3	21.1	17.3	15.5	21.3	13.5
	7.5	22	13.5	14	24	22.5	13	16.5	22
0.5	1.6	1.7	1.8	1.5	1.6	1.7	1.4	1.5	1.6
	0.9	1.1	1.1	0.8	1.3	0.9	0.8	0.8	0.9
	1	1	1	1	1	1	1	1	1
1	1	1	1	1	1	1	1	1	1
	0	0	0	0	0	0	0	0	0
	1	1	1	1	1	1	1	1	1
3	1	1	1	1	1	1	1	1	1
	0	0	0	0	0	0	0	0	0
	1	1	1	1	1	1	1	1	1

**Table 9:** The run length characteristic (ARL, SDRL (in 2nd row) and MDRL (in 3rd row)) of the proposed EWMA-SR and HWMA-SR for different distributions when $n=15$ and $ARL_0=500$.

EWMA-SR Repetitive				HWMA-SR Repetitive		
$\delta$	$\omega =0.03$	$\omega =0.05$	$\omega =0.2$	$\omega =0.03$	$\omega =0.05$	$\omega =0.2$
Normal (0,1)						
0.0	500.4	499.8	500.5	500.8	501.5	500.9
	498.3	471.6	505.8	515.9	454.9	412.1
	359	369	333	403	421	450
0.1	23.5	36.8	53.6	17.2	21.2	23.1
	15.9	25.2	49.1	24.5	16.3	22.7
	20	30	39	8.5	18.5	19.5
0.5	3.4	4.4	5.2	1.4	1.7	1.8
	0.9	1.1	0.8	0.8	0.8	1.1
	3	4	5	1	1.5	1
1	2	2	2	1	1	1
	0.3	0.5	0.8	0	0	0
	2	2	2	1	1	!
3	2	2	2	1	1	1
	0	0	0	0	0	0
	2	2	2	1	1	1
Laplace (0,$1/\sqrt{2}$)						
0.0	503.6	504.0	504.2	501.7	499.1	501.9
	490.3	512.5	501.1	556.8	537.9	542.2
	343.5	340	333.5	395	386	260.5
0.1	14.9	15.7	29.0	8.7	13.4	15.9
	9.2	11.1	25.6	7.1	11.3	16.6
	13	13	21	7	10	7
0.5	3.1	2.5	2.7	1.2	1.2	1.4
	0.8	0.7	0.7	0.6	0.6	0.7
	3	2	2	1	1	1
1	2	2	2	1	1	1
	0.3	0.1	0.4	0	0	0
	2	2	2	1	1	1
3	2	2	2	1	1	1
	0	0	0	0	0	0
	2	2	2	1	1	1
LG (0,$\sqrt{3}/\pi$)						
0.0	501.2	502.3	502.9	505.3	499.3	499.0
	510.9	509.9	496.3	461.1	413.1	362.7
	423.2	340	355	413	380.5	497
0.1	20.4	21.7	45.4	10.1	14.6	22.5
	13.8	16.7	41.6	9.7	16.1	15.4
	17	17	33	5.5	9	20
0.5	3.3	2.6	2	1.2	1.4	1.5
	0.9	0.8	0.7	0.6	0.9	0.8
	3	2	2	1	1	1
1	2	2	2	1	1	1
	0.2	0.2	0	0	0	0
	2	2	2	1	1	1
3	2	2	2	1	1	1
	0	0	0	0	0	0
	2	2	2	1	1	1
Student’s t-Distribution (4)						
0.0	499.0	502.8	500.3	502.2	500.4	500.7
	487.5	504.8	498.7	421.3	488.6	386.5
	357.5	353.5	348	465.5	254.5	362.5
0.1	23.1	34.2	51.2	11.5	15.7	20.3
	17.3	24.6	49.5	13.6	18.5	17.6
	20	28	36	5.5	11.5	17.5
0.5	3.4	2.1	2	1.5	1.6	1.3
	0.9	1.1	0.8	0.8	0.9	0.9
	3	2	2	1	1	1
1	2.1	2.5	2	1	1	1
	0.3	0.5	0	0	0	0
	2	2	2	1	1	!
3	2	2	2	1	1	1
	0	0	0	0	0	0
	2	2	2	1	1	1
Student’s t-Distribution (8)						
0.0	499.4	499.0	501.4	499.2	499.9	507.6
	493.6	511.3	498.3	497.7	476.9	462.8
	348	340	342.5	415	389.5	377.5
0.1	23.6	34.9	53.2	14.4	19.8	21
	18.3	24.9	51.9	14.9	20.9	21.2
	18	27	36.5	11	11.5	11
0.5	3.4	4.1	2.1	1.4	1.4	1.5
	0.9	1.1	0.9	0.8	0.8	0.8
	3	4	2	1	1	1
1	2.1	2.4	2	1	1	1
	0.3	0.5	0	0	0	0
	2	2	2	1	1	1
3	2	2	2	1	1	1
	0	0	0	0	0	0
	2	2	2	1	1	1

## Illustrative example

In this part, an example to observe the zero state is provided to determine the application of the HWMA-SR repetitive chart and EWMA-SR repetitive chart together with the counterpart charts to compare the effectiveness in a non-normal but symmetrical distribution, the Laplace distribution is expanded to the recognition of such situations. The data were achieved by simulating the first 30 subgroups of size 10 each created from the Laplace distribution, for in control process with location=0, scale=$1/\sqrt{(}2)$, under fixed IC $ARL =370$. Presume that the process is altered rising by 0.25; so, the next 20 subgroups of size 10 are made from the Laplace distribution with parameters (location=0.25, scale=1). Same as, we obtained the data for the Auxiliary HWMA-SR repetitive chart by simulating 30 subgroups of size 10, from $(X, Y)\sim N_2 (\mu _X, \mu _Y, \sigma _X, \sigma _Y, \rho$) for IC process with parameters (location=0, scale=$1/\sqrt{2},\rho =0.25$). The afterward 20 subgroups of size 10 values are made after introducing a shift in the process by 0.25, from $(X, Y)\sim N_2 (\mu _X+\delta \sigma _X, \mu _Y, \sigma _X, \sigma _Y, \rho$) with parameters (location=0.25, scale=$1/\sqrt{2}, \rho =0.25$). Data is presented in Table 10.

**Table 10:** The Simulated Data.

S. No	$Z_j$	$H_j$	$M_j$	S. No	$Z_j$	$H_j$	$M_j$
1	0.9 00	0.500	$-$1.525	26	0.332	1.000	$-$8.058
2	$-$2.49	4.600	$-$13.700	27	$-$0.601	$-$2.200	$-$6.931
3	$-$0.341	1.600	$-$4.425	28 $-$	3.041	2.533	$-$2.833
4	$-$1.007	$-$3.000	4.150	29	$-$3.037	$-$1.593	$-$7.562
5	$-$2.406	$-$5.200	1.650	30	$-$0.433	$-$0.331	$-$6.281
6	$-$0.665	$-$6.960	$-$1.480	31	$-$1.900	0.900	$-$0.325
7	0.101	$-$7.100	$-$3.275	32	$-$1.010	4.600	$-$3.250
8	0.591	$-$9.543	$-$2.843	33	$-$0.009	$-$10.600	$-$3.500
9	1.632	$-$10.325	$-$6.168	34	2.292	$-$2.200	$-$1.450
10	$-$3.432	$-$9.000	$-$7.200	35	1.963	1.700	1.425
11	$-$1.388	$-$10.400	$-$11.205	36	3.466	2.960	0.990
12	$-$1.749	$-$9.145	$-$11.432	37	4.619	2.500	3.050
13	2.125	$-$5.100	$-$11.725	38	9.457	2.971	7.800
14	2.613	$-$6.969	$-$9.346	39	9.412	4.225	12.906
15	$-$0.348	$-$4.943	$-$4.975	40	12.171	6.200	14.700
16	$-$3.012	$-$6.480	$-$4.860	41	13.254	10.280	11.755
17	$-$2.212	$-$4.812	$-$5.553	42	9.828	8.218	11.465
18	1.109	$-$5.317	$-$4.115	43	12.545	9.950	13.112
19	$-$1.702	$-$1.300	$-$6.450	44	11.391	10.153	16.285
20	$-$1.831	$-$0.989	$-$7.105	45	10.952	10.700	13.532
21	$-$3.348	2.010	$-$12.812	46	6.956	12.520	15.430
22	$-$0.113	1.171	$-$12.214	47	7.161	11.675	13.368
23	$-$0.402	0.109	$-$9.988	48	6.145	9.435	11.938
24	$-$0.862	0.573	$-$9.263	49	9.631	8.100	12.050
25	0.924	1.875	$-$10.050	50	7.967	8.389	14.015

**Figure 6:**
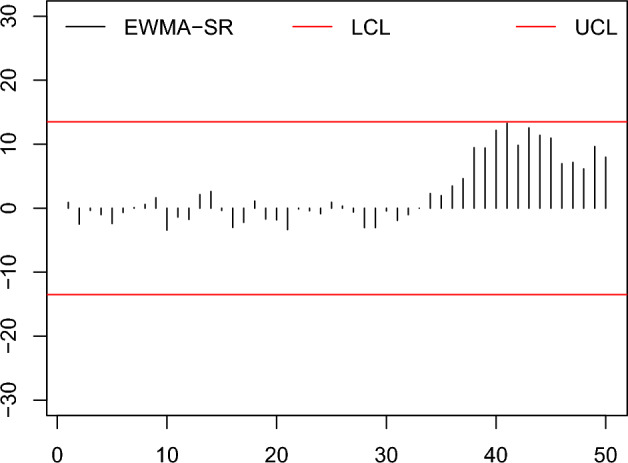
The existing EWMA-SR chart^19^ with designed parameter $\omega =0.10$, $n=10$ and coefficient of control limit (2.792).

**Figure 7:**
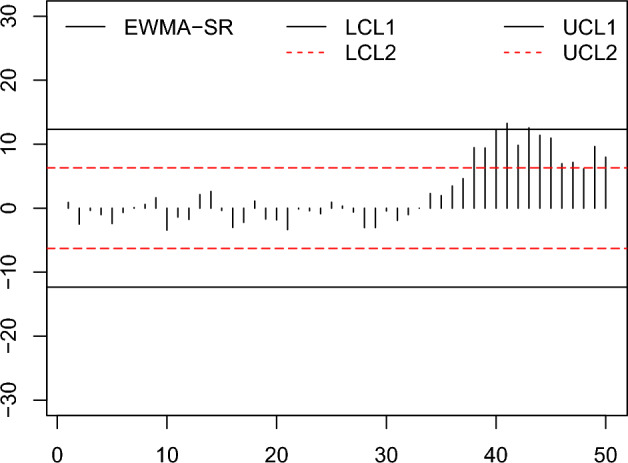
The proposed EWMA-SR repetitive chart with designed parameter $\omega =0.10$, $n=10$ and coefficients of control limit (2.739, 1.399).

**Figure 8:**
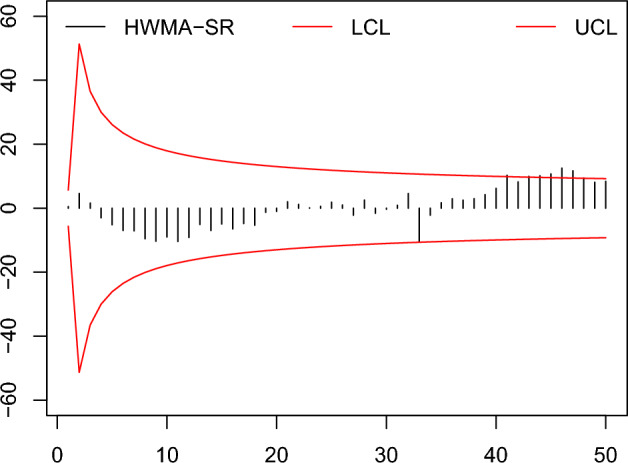
The existing HWMA-SR chart^26^ with designed parameter $\omega =0.10, n=10$ and coefficient of control limit (2.587).

**Figure 9:**
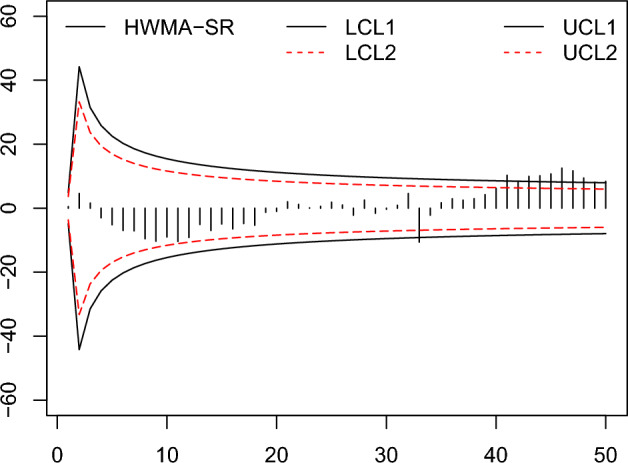
The proposed HWMA-SR repetitive chart with designed parameters $\omega =0.10, n=10$, and coefficients of control limit (2.650, 1.190).

**Figure 10:**
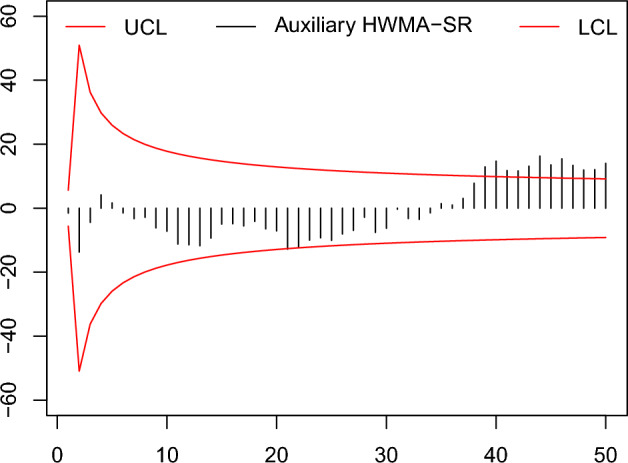
The existing auxiliary HWMA-SR chart with designed parameters $n=10,\omega =0.10, \rho =0.25$ and coefficient of control limit (2.967).

**Figure 11:**
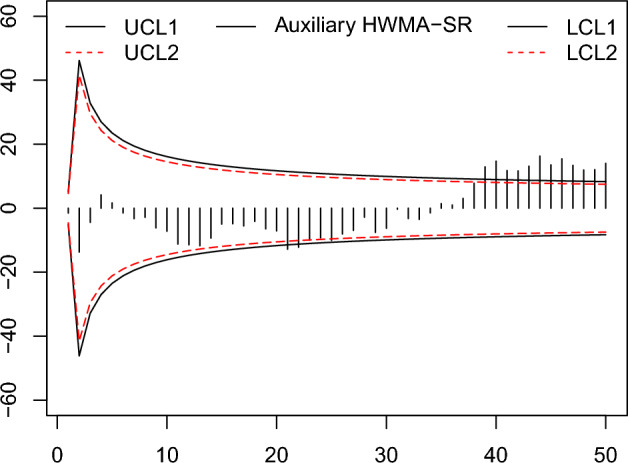
The proposed auxiliary HWMA-SR repetitive chart with designed parameters $n=10, \omega =0.10, \rho =0.25$ and coefficients of control limit (3.202,1.152).

The nonparametric existing EWMA-SR, HWMA-SR, and Auxiliary HWMA-SR charts are also composed of smoothing constant $\omega =0.10, \rho =0.25$ and plotting coefficients 2.792, 2.587, and 2.967, at fixed IC ARL=370. For the sensible calculation, the proposed EWMA-SR repetitive, HWMA-SR repetitive, and Auxiliary HWMA-SR repetitive charts are also constructed with smoothing constant $\omega =0.10, \rho =0.25$ and charting coefficients for EWMA-SR repetitive is (2.739, 1.399), for HWMA-SR repetitive is (2.650, 1.190), and Auxiliary HWMA-SR repetitive is (3.202, 1.152), respectively, at fixed IC $ARL = 370$. The consequential control charts are exposed in Figs. 6, 7, 8, 9, 10, 11.

From the Figs. 6, 7, 8, 9, 10, 11, it is noticed that the proposed charts detect the process shift earlier as compared to counterpart charts. The proposed chart EWMA-SR repetitive and HWMA-SR repetitive charts identify the OOC values at sample numbers 40 and 32; however, the existing charts EWMA-SR chart doesn’t detect any OOC values at any point, but HWMA-SR declares the OOC process at sample number 45. The current chart Auxiliary HWMA-SR claims the first OOC signal at sample number 38, separately. So, the existing charts EWMA-SR, HWMA-SR, and Auxiliary HWMA-SR require 0, 15, and 8 samples, respectively, taxable the earliest OOC signal once the existence of the process shift. But the plotted Auxiliary HWMA-SR repetitive chart, displayed in Fig. 11, reveals the process at sample number 21. Instead, the offered Auxiliary HWMA-SR repetitive chart has taken the OOC signal before the existence of the shift. Due to the fast detection ability of the proposed charts (EWMA-SR repetitive, HWMA-SR repetitive, and Auxiliary HWMA-SR repetitive), they give a total of 1, 8, and 14 OOC signals as compared to the existing charts (EWMA-SR, HWMA-SR, and Auxiliary HWMA-SR) OOC signals 0, 2, and 12, respectively. The results confirm this, and the proposed charts perform well overall than the existing charts. Other than that, the proposed chart Auxiliary HWMA-SR repetitive chart performs well compared to all other charts considered in this manuscript.

## Decision and future recommendations

In this study, a new well-organized nonparametric control chart proposes for examining a small shift in the process mean where the process variable is linked with an auxiliary variable using a repetitive sampling scheme. The proposed chart is based on HWMA customs, both the process and auxiliary variable, to form a regression estimator that revenues a well-organized and impartial estimate of the process mean. Other than that, we also propose a nonparametric or distribution-free EWMA and HWMA chart using repetitive sampling and compare them with an auxiliary HWMA chart under the repetitive scheme. The performance of the proposed charts is provided in terms of its run length properties under different symmetric distributions by using the Monte Carlo Simulation. The comparison has recognized the power of the proposed charts EWMA-SR repetitive, HWMA-SR repetitive, and Auxiliary HWMA-SR repetitive over the counterpart control charts supposed here. The justification behind the EWMA and HWMA statistics was to allot weights to the observations. The new sample gets more influence, decreasing exponentially as the sample goes less contemporary. In the field of modern technology, we always try to find more efficient ways to get excellent quality products in a short time and less expensive. This study proposed an improvement over the improved EWMA, HWMA, and Auxiliary HWMA control charts, so-called EWMA-RS, HWMA-RS, and AHWMA-RS control charts. The speculative properties of the proposed statistics are originated and the control limits structure is formulated. The performance evaluation of the proposed charts is done through extensive simulations and comparison with other control charts showing the efficiency zones to the newly developed charts over the existing charts. The achievement of the proposed charts is verified using a manufacturing process.

The simulation study revealed that the chart identifies a shift in the process quicker than other charts and methods. Moreover, the ARL contrasts of all the existing charts showed that the proposed charts are more efficient and quickly detect the shift in the process mean. For example, the EWMA-SR with a repetitive sampling scheme performs the best as compared to a single sampling scheme, it is noticed that, at $\delta =0.25, \omega =0.05$, and $n=10$, the $ARL_1$ values are 11.2, 8.3,10.1, and 11.5 for repetitive sampling with different distributions and the $ARL_1$ values are 16.8, 12.7, 15.3, and 13.1 for single sampling scheme with different distributions. When we check the performance of both schemes for the HWMA-SR chart, we also noticed that the RS scheme performs the best as compared to the SS scheme. For example, at $\delta =0.5,\omega =0.05$, and $n=10$, the $ARL_1$ values for the RS scheme are 1.5,1,1,1.4 and the $ARL_1$ values for the SS scheme are 11.8,8.2,10.4,8.7. The comparison uncovers that EWMA-SR repetitive chart performs the best at the different levels of shifts plus the different levels of smoothing parameter ($\omega$) with $n=10$. For example, when $\omega =0.03, n=10$, and $\delta =0.10,0.5,1.0,2.0, and 3.0$, the $ARL_1$ values for the proposed chart are 29.5, 3.7, 2.2, 2, 2, and 2, whereas the $ARL_1$ for the existing chart are 59.6, 8.5, 5.2, 4, 4, and 4 (cf. Table 7). From Table 7 results, it is observed that with all selections of designed parameters, the proposed chart performs more efficiently as compared to the existing chart. Moreover, these results show that the proposed chart is better in terms of detection ability at all levels of shifts than the existing chart.

The proposed chart and existing chart $ARL_1$ values are compared in Table 9 at a different level of shifts. So, the results have shown that the proposed chart has significantly better performance as compared to the counterpart chart. For instant, with $n=10, \omega =(0.03,0.05,0.1$, and 0.2), and $\delta =0.1$, the proposed chart $ARL_1=13.2, 15.4, 17.9, 21.3$ whereas the corresponding $ARL_1=44.8, 48.5, 52.1, 52.9$ for HWMA-SR chart. From these results, we noticed a considerably improved performance of the HWMA-SR repetitive chart as compared to the HWMA-SR chart. Moreover, we also noticed that when $\omega$ increases the $ARL_1$ also increases at the same level of shift and sample size. The same trend was also noticed in all other charts.

The run-length acatalectics values of the proposed chart are reported in Table 7 and it is observed that the $ARL_1$ values of the proposed chart are less than the Auxiliary HWMA-SR chart, under all shifts in the process. Moreover, we also have seen the decreasing trend at all levels of the shift in the $ARL_1$ values when $\rho$ values increase and $ARL_1$ values increased when $\omega$ values increase. For example, when $n=10, \omega =0.10, 0.1$ and $\rho =(0.05, 0.25, 0.5, 0.75)$, the $ARL_1$ values for Auxiliary HWMA-SR repetitive chart are 13, 12.7, 12.5, and 11.5 against the $ARL_1$ values for the existing chart are 52.1, 44.5, 39.6, and 36.3. Furthermore, when $n=10, \rho =0.50$, and $\omega =(0.03,0.05, 0.1, 0.2)$, the $ARL_1$ values for the proposed chart are 9.8, 11.6, 12.2, and 17.2 while the $ARL_1$ values for the existing chart are 27.7, 38.7, 39.5, and 44.7. The proposed charts are more efficient as compared to the existing charts because the proposed charts detect the out-of-control signal earlier. So, the proposed charts can be used in industries for the production of various quality products by saving money and time. The scope of the proposed chart can be investigated by using different nonparametric tests and other well-known sampling techniques.

### Supplementary Information

Supplementary Information.

## Data Availability

The data supporting this study’s findings are simulated data set (self-generated data set) details available in the illustrative example of this article.
