# Supplementary material for: Efficient signed-rank based EWMA and HWMA repetitive control charts for monitoring process mean with and without auxiliary information
Source: Sci Rep. 2023 Sep 30;13:16459. doi: 10.1038/s41598-023-42632-x (PMC10542769; doi:10.1038/s41598-023-42632-x)
Supplement: Supplementary file 1 — Supplementary Information. [file 41598_2023_42632_MOESM1_ESM.pdf]

1. Appendix A

A.1

We have applied the expectation on both sides of Eq. 4.

$$E(H_j) = E(\omega SR_{X_j} + (1-\omega)\overline{SR}_{X_{j-1}})$$
$$E(H_j) = E(\omega SR_{X_j} + \frac{(1-\omega)}{j-1}\Sigma_{i=1}^{j-1} SR_{X_i}$$
$$E(H_j) = \omega E(SR_{X_j}) + E(\frac{(1-\omega)}{j-1}\Sigma_{i=1}^{j-1} SR_{X_i})$$
$$E(H_j) = \omega\theta_0 + (1-\omega)\theta_0$$
$$E(H_j) = \theta_0(\omega + 1 - \omega)$$
$$E(H_j) = \theta_0 = 0$$

the Variance of H_j can be obtained by solving Eq. 4 as:

$$Var(H_j) = Var(\omega SR_j + (1-\omega)\overline{SR}_{j-1})$$
$$Var(H_j) = \omega^2 Var(SR_j) + (1-\omega)^2 Var($$
$$\overline{SR}_{j-1}) + \omega(1-\omega)Covar(SR_j, \overline{SR}_{j-1})$$
$$Var(H_j) = \begin{cases} \frac{\omega^2 n(n+1)(2n+1)}{6}, & \text{if} j = 1 \\ \frac{n(n+1)(2n+1)}{6}(\omega^2 + \frac{(1-\omega)^2}{j-1}), & \text{if} j > 1 \end{cases}$$

For more detail, we referred to the reader to read Appendix A.2 Abbas [19] .

A.2.

We have applied the expectation on both sides of Eq. 13.

$$E(M_j) = E(\omega SR_{Y_j^*} + (1-\omega)\overline{SR}_{Y_{j-1}^*})$$
$$E(M_j) = E(\omega SR_{Y_j^*} + \frac{(1-\omega)}{j-1}\Sigma_{i=1}^{j-1} SR_{Y_i^*})$$
$$E(M_j) = \omega E(SR_{Y_j^*}) + E(\frac{(1-\omega)}{j-1}\Sigma_{i=1}^{j-1} SR_{Y_i^*})$$
$$E(M_j) = \omega\theta_0 + (1-\omega)\theta_0$$
$$E(M_j) = \theta_0(\omega + 1 - \omega)$$
$$E(M_j) = \theta_0 = 0$$

the Variance of M_j can be obtained by solving Eq.13 as:

$$Var(M_j) = Var(\omega SR_{Y_j^*} + (1-\omega)\overline{SR}_{Y_{j-1}^*})$$
$$Var(H_j) = \omega^2 Var(SR_{Y_j^*}) + (1-\omega)^2 Var($$
$$\overline{SR}_{Y_{j-1}^*}) + \omega(1-\omega)Covar(SR_{Y_j^*}, \overline{SR}_{Y_{j-1}^*})$$
$$Var(H_j) = \begin{cases} \frac{\omega^2 n(n+1)(2n+1)}{6}, & \text{if} j = 1 \\ \frac{n(n+1)(2n+1)}{6}(\omega^2 + \frac{(1-\omega)^2}{j-1}), & \text{if} j > 1 \end{cases}$$

2. R code

```
arl=c();sdt=c() ; mdn=c(); perc25=c(); perc75=c(); w103=perc90=c()
```

CL = UCL1 = LCL1=UCL2=LCL2= double();sigy=sigx=1;rho=0.25;n=5;mux=muy=mu0=0;w=0.03;ybar=c();rp=10;s[illegible]
H=SR=W=v=c()

#set.seed(5566)
k1=2.269
k2=0.554
sdt=(n*(n+1)*(2*n+1))/6;sdt
# Regresion parameters mu0=muy; s0 = sqrt($(sigy^2) * (1 - rho^2)$)
covxy=$rho * sigy * sigx; beta = covxy/sigx^2$
shift=c(0, 0.05, 0.1, 0.15, 0.25, 0.5, 1, 2,3)
for(b in 1:length(shift))
for(p in 1:rp)
for(i in 1:ss)
if(i==1)
LCL1[i]=$mu0 - k1 * sqrt(w^2 * sdt)$
$UCL1[i] = mu0 + k1 * sqrt(w^2 * sdt)$
$LCL2[i] = mu0 - k2 * sqrt(w^2 * sdt)$
$UCL2[i] = mu0 + k2 * sqrt(w^2 * sdt)$
else
$tt = w^2 + (1 - w)^2 * 1/(i - 1)$
$LCL1[i] = mu0 - k1 * sqrt(tt * sdt)$
$UCL1[i] = mu0 + k1 * sqrt(tt * sdt)$
$LCL2[i] = mu0 - k2 * sqrt(tt * sdt)$
$UCL2[i] = mu0 + k2 * sqrt(tt * sdt)$

runs=0
rep=0
mu = muy + shift[b]*(sigy)
z1 = rnorm(n); z2 = rnorm(n)
# Z1=rlaplace(n,shift,1/sqrt(2))
#Z2=rlaplace(n,shift,1/sqrt(2))
#rlogis(n,p1,sqrt(3)/pi))
#rt(n,p1,1)
x = $sqrt(1 - rho^2) * sigx * z1 + rho * sigx * z2 + mux; y = sigy * z2 + mu$
$ybar = y + beta * (mux - x)\#regressionequation$
$v < -wilcox.test(ybar, mu = 0)$
$W = v\$statistic$
$SR[i] = 2 * W - (n * (n + 1)/2)$
if(i==1)
H[i]=w*SR[i]
else
for(j in i:1)
if(j==i)
H[i]=w*SR[i]
else
H[i]=H[i]+(1-w)/(i-1) * mean(SR[j])

if(H[i]>UCL1[i] | H[i]<LCL1[i])
runs=i

if((H[i]>=LCL1[i] & H[i]<LCL2[i]) | (H[i]>UCL2[i] & H[i]<=UCL1[i]))
rep=rep+1;

```
if(runs>0)
RL[p]=runs-rep
break;



arl[b]=mean(RL)

print(cbind(arl,shift))
# HWMA control chart Code
y=c();hwma=c();ucl1=lcl1=c();ARL=c();
lcl2=ucl2=c();a=c();n=1;ld=0.03; L1=2.338;L2=1.803;
mu0=0; sig0=1;
shift=c(0, 0.05, 0.075, 0.1, 0.125, 0.15, 0.175, 0.2, 0.25, 0.5, 0.75, 1, 1.5, 2,3)
for(b in 1:length(shift))
shift=p
for(j in 1:300)

rep=0;runs=0;
for(i in 1:100000)

y[i]=(rnorm(n,p,1)-mu0)/sig0
#rlaplace(n,shift,1/sqrt(2))
#rlogis(n,p1,sqrt(3)/pi))
#rt(n,p1,1)
if(i==1)hwma[i]=ld*y[i]elsehwma[i]=ld*y[i]+((1-ld)*mean(y[1:(i-1)]))
if(i==1)
ucl1[i]=mu0 + L1 * sqrt((ld^2 * sig0^2)/n)
lcl1[i] = mu0 - L1 * sqrt((ld^2 * sig0^2)/n)

if(i>1)
ucl1[i]=mu0 + L1 * sqrt((ld^2 * sig0^2)/n + ((1 - ld)^2 * sig0^2)/(n * (i - 1)))
lcl1[i]=mu0 - L1 * sqrt((ld^2 * sig0^2)/n + ((1 - ld)^2 * sig0^2)/(n * (i - 1)))

if(i==1)
ucl2[i]=mu0 + L2 * sqrt((ld^2 * sig0^2)/n)
lcl2[i] = mu0 - L2 * sqrt((ld^2 * sig0^2)/n)

if(i>1)
ucl2[i]=mu0 + L2 * sqrt((ld^2 * sig0^2)/n + ((1 - ld)^2 * sig0^2)/(n * (i - 1)))
lcl2[i] = mu0 - L2 * sqrt((ld^2 * sig0^2)/n + ((1 - ld)^2 * sig0^2)/(n * (i - 1)))

if(hwma[i]>ucl1[i]|hwma[i]<lcl1[i])
runs=i;
break;
if((hwma[i]>=lcl1[i] & hwma[i]<lcl2[i]) | (hwma[i]>ucl2[i] & hwma[i]<=ucl1[i]))
rep=rep+1;


if(runs>0)
a[j]=runs-rep
```

```
ARL[b]=mean(a)

print(cbind(ARL,shift))
#graphical presentation
# the ARL values for all the distributions with different sample size.
plot<-c(); lines<-c();
#ARL=500 for normal distribution
d5E<-log(c(500.1,204.0,67.9,29.2,15.8,10.3,8.1,5.4,4.2,2.3,2,2,2))
d10E<-log(c(503.7,53.5,17.9,5.0,3.2,4.1,2.4,1.5,1.5,1,1,1,1))
d15E<-log(c(501.7,48.1,13.0,5.0,2.9,2.5,2.2,1.7,1,1,1,1,1))

    #lambda=0.10; r0=500;Laplace:
d51E<-log(c(499.0,133.5,38.7,17.4,10.5,7.5,5.9,4.3,3.5,2.3,2,2,2))
d101E<-log(c(504.5,43.8,14.1,4.5,3.6,3.6,1.7,1.3,1.2,1,1,1,1))
d151E<-log(c(499.4,41.4,7.7,3.1,2.8,1.9,1.6,1.2,1,1,1,1,1))

    #lambda=0.15;r0=500;LG:
d15<-log(c(502.7,183.3,58.1,25.1,14.1,9.4,7.1,5.0,4,2.3,2,2,2))
d110<-log(c(500.6,50.9,14.5,4.9,3.9,3.6,1.9,1.6,1.5,1,1,1,1))
d115<-log(c(502.8,45.5,11.8,3.7,3.0,1.8,1.6,1.5,1,1,1,1,1))

    #lambda=0.20;r0=500; student t(8)
d52<-log(c(501.6,203.4,71.0,31.7,17.0,11.2,8.6,5.6,4.2,2.4,2,2,2))
d102<-log(c(501.0,44.0,14.2,4.6,3.5,3.4,1.8,1.5,1.4,1,1,1,1))
d152<-log(c(502.1,42.3,11.8,4.4,2.6,1.9,1.4,1.4,1,1,1,1,1))

    #lambda=0.25; r0=500;student t(4)
d55<-log(c(503.8,211.4,68.8,29.8,16.4,11.1,8.2,5.4,4.2,2.4,2,2,2))
d105<-log(c(503.2,51.9,14.5,4.9,3.9,3.6,1.9,1.6,1.5,1,1,1,1))
d155<-log(c(502.1,47.6,12.4,4.5,3,2.1,1.8,1.6,1.2,1,1,1,1))
layout(matrix(c(1,2,3,4,5,0), 3, 2, byrow = TRUE))
# plot code for all the distribution that showed in the Fig 2 to 4.
x<-c(-3,-2,-1.5,-1.0,-0.5,-0.4,-0.3,-0.25,-0.20,-0.15,-0.10,-0.05,0.00,0.05,0.10,0.15,0.20,0.25,0.3,0.4,0.5,1.0,1.5,2,3)
plot(x,d5E,col="1",pch=1,type="o",lty=2, ylim = c(0.00,7.00), ylab="log(ARL)", xlab=expression(delta), main=
expression(paste (normal(0,1))))
points(p,d10E, type="o", col="2", pch=2,lty=2)
points(p,d15E,type="o", col="3",pch=3,lty=2)

    legend("topright",legend=expression("EWMA-SR","HWMA-SR","Auxiliary HWMA-SR"),bty="n",col=1:3,pch=1:9,lty=
p<-c(0.00,0.05,0.10,0.15,0.20,0.25,0.3,0.4,0.5,1.0,1.5,2,3)
plot(p,d51E,col="1",pch=1,type="o",lty=2, ylim = c(0.00,7.00), ylab="log(ARL)", xlab=expression(delta), main=
expression(paste (Laplace(0,1/sqrt(2)))))
points(p,d101E, type="o", col="2", pch=2,lty=2)
points(p,d151E,type="o", col="3",pch=3,lty=2)

    legend("topright",legend=expression("EWMA-SR","HWMA-SR","Auxiliary HWMA-SR"),bty="n",col=1:3,pch=1:9,lty=
p<-c(0.00,0.05,0.10,0.15,0.20,0.25,0.3,0.4,0.5,1.0,1.5,2,3)
plot(p,d15,col="1",pch=1,type="o",lty=2, ylim = c(0.00,7.00), ylab="log(ARL)",xlab=expression(delta), main= ex-
pression(paste (LG(0,sqrt(3)/pi))))
points(p,d110, type="o", col="2", pch=2,lty=2)
points(p,d115,type="o", col="3",pch=3,lty=2)

    legend("topright",legend=expression("EWMA-SR","HWMA-SR","Auxiliary HWMA-SR"),bty="n",col=1:3,pch=1:9,lty=
```

p<-c(0.00,0.05,0.10,0.15,0.20,0.25,0.3,0.4,0.5,1.0,1.5,2,3)
plot(p,d52,col="1",pch=1,type="o",lty=2, ylim = c(0.00,7.00), ylab="log(ARL)",xlab=expression(delta), main= expression(paste (t(4))))
points(p,d102, type="o", col="2", pch=2,lty=2
) points(p,d152,type="o", col="3",pch=3,lty=2)

legend("topright",legend=expression("EWMA-SR","HWMA-SR","Auxiliary HWMA-SR"),bty="n",col=1:3,pch=1:9,lty=[illegible]
p<-c(0.00,0.05,0.10,0.15,0.20,0.25,0.3,0.4,0.5,1.0,1.5,2,3)
plot(p,d55,col="1",pch=1,type="o",lty=2, ylim = c(0.00,7.00), ylab="log(ARL)",xlab=expression(delta), main= expression(paste (t(8))))
points(p,d105, type="o", col="2", pch=2,lty=2)
points(p,d155,type="o", col="3",pch=3,lty=2)
legend("topright",legend=expression("EWMA-SR","HWMA-SR","Auxiliary HWMA-SR"),bty="n",col=1:3,pch=1:9,lty=2)
